# Effective Theories for Incompressible Magnetoelastic Shallow Shells

**DOI:** 10.1007/s00032-026-00433-7

**Published:** 2026-04-28

**Authors:** Emanuele Tasso, Tobias Unterberger

**Affiliations:** Institute of Analysis and Scientific Computing, TU Wien, Wiedner Hauptstraße 8-10, 1040 Vienna, Austria

**Keywords:** $\Gamma$-convergence, Magnetoelasticity, Dimension reduction

## Abstract

We characterize the asymptotic behaviour, in the sense of $\Gamma$-convergence, of a thin magnetoelastic shallow shell. The compactness is achieved up to rigid motions. For deformations, it relies on an approximation by rigid movements, whereas for magnetizations it is based on a careful consideration of the geometry of the deformed domain. This result is a generalization of [1] by incorporating geometric effects due to vanishing curvature, which constitute the main novelty of the analysis.

## Introduction

The rigorous derivation of reduced models for thin structures by means of $\Gamma$-convergence is by now a classical topic in nonlinear elasticity; see, for instance, [10] and the references therein. These techniques have led to a detailed understanding of plates, rods, and shells in purely elastic settings, where geometric effects such as bending and curvature play a central role in the effective lower-dimensional theories.

In contrast, the identification of reduced models in *magnetoelasticity* remains substantially more challenging, especially in the regime of large strains. The main difficulty stems from the intrinsic coupling between mechanics and magnetism: elastic energies are naturally formulated in Lagrangian coordinates, whereas magnetic energies are Eulerian and typically nonlocal. This mixed Euler-Lagrangian structure obstructs a direct application of standard compactness and dimension-reduction arguments. At present, the only available $\Gamma$-convergence results for incompressible magnetoelastic bodies in the large-strain setting are due to M. Bresciani and collaborators, who derived effective plate theories in a series of works [1, 3, 4].

The purpose of the present paper is to extend this line of research from flat plates to the geometrically richer setting of *magnetoelastic shallow shells*.

Shallow shells can be informally described as thin elastic bodies whose midsurface is a small geometric perturbation of a flat domain. They can be seen as intermediate objects between flat plates [5] and general shells [6], retaining many features of plates while incorporating mild curvature effects. Although the curvature of the midsurface vanishes as the thickness tends to zero, experience from elasticity theory suggests that even such small geometric deviations may influence the effective two-dimensional behavior [15, 17].

The central question addressed in this work is whether the magnetoelastic plate theory remains valid in this shallow shell regime, or whether the underlying geometry leaves a detectable trace in the limiting two-dimensional model.

To make this precise, let $\Omega = \omega \times (-\tfrac{1}{2},\tfrac{1}{2})$ with $\omega \subset \mathbb {R}^2$. For $h>0$, the associated plate is $\Omega _h = \omega \times (-\tfrac{h}{2},\tfrac{h}{2})$, while the corresponding shallow shell $\hat{\Omega }_h$ is defined as the image of $\Omega$ under the mapping

$$ (x',x_3) \mapsto (x', h\theta (x')) + h x_3 n_h(x'), $$

where $\theta$ is a smooth function and $n_h$ denotes the unit normal to the midsurface of $\hat{\Omega }_h$. This construction describes a family of thin three-dimensional bodies whose reference configuration approaches a flat plate as $h \rightarrow 0$, while retaining information on the underlying geometry.

The mechanical response of the shell is described by an incompressible deformation $u_h: \hat{\Omega }_h \rightarrow \mathbb {R}^3$, coupled with a magnetization $m_h: u_h(\hat{\Omega }_h) \rightarrow \mathbb {S}^2$. The total energy consists of three competing contributions: a nonlinear elastic energy enforcing incompressibility, a local magnetic exchange energy favoring alignment of magnetic moments, and a nonlocal magnetostatic energy accounting for long-range interactions. More precisely, we consider the functional

1.1 $$\begin{aligned} \begin{aligned}&\mathcal {E}_h(u_h,m_h) := \frac{1}{h^\beta }\int _{\hat{\Omega }_h} W^{\textrm{inc}}(\nabla u_h, m_h \circ u_h)\, dX \\&\quad + \alpha \int _{u_h(\hat{\Omega }_h)} |\nabla m_h|^2 \, d\xi + \frac{1}{2} \int _{\mathbb {R}^3} |\nabla \psi _{m_h}|^2 \, d\xi , \end{aligned} \end{aligned}$$

where $\beta >2p$ and $p>3$. The incompressible elastic density $W^{\textrm{inc}}$ enforces the constraint $\det \nabla u_h = 1$, while the stray-field potential $\psi _{m_h}$ solves the Maxwell equation

$$ \Delta \psi _{m_h} = \textrm{div}(\chi _{\hat{\Omega }_h^{u_h}} m_h) \quad \text {in } \mathbb {R}^3. $$

Precise assumptions on the elastic energy density and on the admissible configurations are specified in Sect. 3.

The main contribution of this work is the characterization of the asymptotic behavior of the rescaled energies $h^{-1}\mathcal {E}_h$ in the thin-film limit. We show that these functionals $\Gamma$-converge to an effective two-dimensional energy depending on in-plane and out-of-plane displacements and on a limiting magnetization. In contrast to the plate model of [1], the limiting functional retains explicit information on the shell geometry, even though the curvature vanishes asymptotically. This highlights geometry as the principal new ingredient of the analysis. The main result, in a simplified statement, reads as follows.

### Theorem 1.1

The asymptotic behaviour of the functionals $(h^{-1}\mathcal {E}_h)$, as $h \rightarrow 0^+$, is described, in the sense of $\Gamma$-convergence, by the functional:

1.2 $$\begin{aligned} E(u,v,\lambda ) := \  &\frac{1}{2}\int _\omega Q_2^{inc}(\text {sym}(\nabla ' u + \nabla 'v \otimes \nabla '\theta ), \lambda ) \ dx' + \frac{1}{24}\int _\omega Q_2^{inc}((\nabla ')^2v,\lambda ) \ dx' \nonumber \\ + \  &\alpha \int _\omega |\nabla '\lambda |^2 \ dx' + \frac{1}{2}\int _\omega |(\lambda )^3|^2 \ dx', \end{aligned}$$

defined for $u \in W^{1,2}(\omega ;\mathbb {R}^2)$, $v \in W^{2,2}(\omega )$ and $\lambda \in W^{1,2}(\omega ;\mathbb {S}^2)$. Given $\nu \in \mathbb {S}^2$, the function $Q_2^{inc}$ is a quadratic form on $\mathbb {R}^{2\times 2}$ constructed from the second-order approximation of $W(.,\nu )$ close to the identity. $\nabla '$ denotes the gradient with respect to the variable $x' \in \omega$ and $(\lambda )^3$ is the third component of $\lambda$.

Here, *u* and *v* represent the in-plane and out-of-plane displacements, respectively, while $\lambda$ denotes the limiting magnetization. The quadratic form $Q_2^{\textrm{inc}}$ is obtained from the second-order expansion of the three-dimensional elastic energy around the identity. The geometric coupling term

$$ \nabla ' v \otimes \nabla ' \theta $$

encodes the interaction between the out-of-plane deformation *v* and the pre-existing slope of the midsurface $\theta$. Even as the curvature of the shallow shell vanishes in the thin-shell limit, this term captures the first-order influence of the shell’s geometry on the in-plane strain.

Intuitively, bending the shell along the direction of the midsurface slope induces in-plane stretching or compression, so that the effective two-dimensional magnetoelastic response differs from that of a flat plate. This term therefore represents the leading-order geometric memory of the original shell in the reduced model, highlighting how even mild curvature modifies the limiting behavior of the system.

### Strategy of the Proof

While our analysis builds upon well-established ideas from the theory of $\Gamma$-convergence and nonlinear elasticity, the contribution of the present work lies in the systematic transfer of magnetoelastic plate models to the geometrically richer setting of shallow shells, together with a careful treatment of the magnetic contributions under minimal structural assumptions.

A central observation underlying our approach is that shallow shells can be regarded as smooth perturbations of flat plates, in the sense that the mapping from the reference plate domain $\Omega _h$ to the shallow shell $\hat{\Omega }_h$ is a $C^1$-diffeomorphism. This geometric property allows us to reduce the analysis of the three-dimensional magnetoelastic energy on shells to the corresponding plate problem, provided that the relevant compactness and lower semicontinuity properties are stable under this transformation. Establishing this stability in the presence of incompressibility and magnetic effects is the central work of the paper.

*Compactness.* The compactness and lower bound results are collected in Theorem 3.4. Their proof relies on an approximation of the deformation gradients by rotation-valued Sobolev maps, extending the classical rigidity framework of Friesecke, James, and Müller [13] to the growth assumptions relevant for magnetoelastic materials (cf. (W.3)). While analogous results are available for purely elastic plates with quadratic growth [15, 18], additional difficulties arise here due to the coupling with magnetization and the nonlinear geometry of the deformed configurations. In particular, compactness of the magnetization requires a refined geometric analysis of the deformed domains: by combining uniform convergence estimates for the deformations with elementary properties of the topological degree, we show that the images $u_h(\Omega _h)$ contain cylinders of height proportional to *h*. This geometric insight allows us to recover compactness of the magnetization via standard arguments on these cylinders and to subsequently identify the limiting behavior globally; see [1, Proposition 4.2] for related ideas.

*Lower-bound.* The lower bound for the energy is established by exploiting the reduction to the plate setting. For the elastic contribution, we adapt dimension-reduction techniques from nonlinear elasticity [12], incorporating incompressibility along the lines of [8, 16]. The magnetic exchange and magnetostatic energies are treated by combining the geometric description of the deformed domains with suitable adaptations of compactness and convergence results for thin magnetic films [14]. This unified approach allows us to recover the full limiting energy without imposing additional regularity assumptions on the magnetization.

*Upper-bound.* The matching upper bound is addressed in Theorem 3.5. Here, we construct recovery sequences by suitably adapting known ansätze for shallow shells [18] and incorporating incompressibility via techniques developed in [16]. The resulting deformations are small perturbations of the identity and are globally injective by a result of [7]. Once again, the $C^1$-diffeomorphic correspondence between plates and shallow shells plays a crucial role in transferring the construction from the plate setting to shells. Convergence of the elastic, exchange, and magnetostatic energies then follows by arguments parallel to those used for the lower bound.

### Outline

In conclusion, starting from existing dimension-reduction results in nonlinear elasticity and magnetoelasticity, this work provides a rigorous derivation of an effective two-dimensional theory for incompressible magnetoelastic shallow shells. The framework developed here suggests several directions for future research, including the treatment of general shell geometries, where curvature effects may become dominant, and extensions to $W^{1,p}$-deformations with $p \ge 2$, which would require more delicate measure-theoretic tools [2]. Moreover, anisotropic exchange interactions as well as DMI terms [11] could be incorporated without substantial changes, as they enter the energy as continuous perturbations.

## Notation and Preliminary Results

In this section we introduce the relevant notation as well as we recall some results which are used in the proceeding sections.

### Notation

- By $B_r(x)$ we denote the ball of radius *r* centered at *x*. If $x \in \mathbb {R}^2$ we write $D_r(x):= B_r(x)$.
- Given a vector $x = (x_1, x_2, x_3) \in \mathbb {R}^3$, we write $x' = (x_1, x_2)$ and also $(x)^3$ for the third component.
- By $\pi$ we denote the projection $\pi : x \mapsto x'$.
- By $\mathbb {S}^2$ we denote the unit sphere.
- For $x \in \mathbb {R}$, $\bigl \lceil x \bigr \rceil$ denotes the ceiling function of *x*, which maps *x* onto the smallest integer greater or equal to *x*.
- For a $n\times n$-matrix $A \in \mathbb {R}^{n\times n}$ we denote its transpose by $A^T$, its inverse by $A^{-1}$ and its transposed inverse by $A^{-T}$.
- We write $\nabla _h:= (\partial _{x_1}, \partial _{x_2}, \frac{1}{h}\partial _{x_3})^T$ and $\nabla _xg(x,z)$ to indicate that the gradient is taken with respect to *x*.
- If a constant *C* is written as *C*(*A*) it is to explicitily highlight its dependence on *A*.
- We write $\min \{a,b\}:= a \wedge b$ and $\max \{a,b\}:= a \vee b$.
- Strong convergence is denoted by $\rightarrow$ and weak convergence is denoted by $\rightharpoonup$.
- By $\mathcal {L}^n(E)$ we denote the n-dimensional Lebesgue measure of some $E \subseteq \mathbb {R}^n$.
- For the map defining the shallow shell $\Theta _h \circ z_h$ on $\Omega \subseteq \mathbb {R}^3$ later defined in Definition 2.4, we use upper case variables *X*, *Y* and *Z* to denote elements of $\Theta _h(z_h(\Omega ))$ to emphasize the difference in domain compared to $x \in \Omega$.
- Elements of the deformed configuration $\Omega ^u$, as defined in (2.1) are denoted by $\xi$.

### Deformed Configuration

As the deformations *v* are not homeomorphisms, their image is not necessarily open. Also, deformations are assumed to be Sobolev maps and are therefore defined almost everywhere. This possibly leads to ambigiuities of functions defined on the image of the deformation *v*. To solve these issues the following notion of a deformed configuration is used.

#### Definition 2.1

*(Deformed configuration).* Let $\Omega \subseteq \mathbb {R}^n$ be open and bounded and let $v \in C^0(\overline{\Omega };\mathbb {R}^n)$. The deformed configuration $\Omega ^v$, relative to the map *v*, is defined as

2.1 $$\begin{aligned} \Omega ^v := v(\Omega )\backslash v(\partial \Omega ). \end{aligned}$$

#### Lemma 2.2

Let $\Omega \subseteq \mathbb {R}^n$ be open, bounded, and connected. Let $v \in C^0(\overline{\Omega };\mathbb {R}^n)$ be almost everywhere injective. Then $\Omega ^v$ is an open set.

The proof of this result can be found in [1, Lemma 2.1].

### Rigidity Estimate

A fundamental tool in our analysis is the following well known rigidity estimate (cf. [9]).

#### Theorem 2.3

(Rigidity estimate). Let $\Omega \subseteq \mathbb {R}^n$ be a bounded Lipschitz domain, where $n\ge 2$ and $1< p < \infty$. Then, there exists a constant $C(\Omega , n, p) > 0$, such that for all $v \in W^{1,p}(\Omega ;\mathbb {R}^n)$, there exists $R \in SO(n)$, such that

$$\begin{aligned} \left\| {\nabla v - R}\right\| _{L^p(\Omega ;\mathbb {R}^n)}^p \le C(\Omega , n, p)\int _{\Omega }  dist ^p(\nabla v , SO(n)) dx. \end{aligned}$$

In particular, *C* is invariant under dilations of $\Omega$, and it is also uniform for the uniform bilipschitz images of a unit ball in $\mathbb {R}^n$.

### Shallow Shells

For $\omega \subseteq \mathbb {R}^2$ bounded, connected Lipschitz domain we denote the corresponding plate with thickness 1 by $\Omega = \omega \times (-\frac{1}{2},\frac{1}{2})$. Call $\Omega _h = z_h(\Omega ):= \omega \times (-\frac{h}{2},\frac{h}{2})$ the plate with thickness *h*. Given a function $\theta \in C^\infty (\overline{\omega })$, we consider the map $\Theta _h: \overline{\Omega }_h \rightarrow \mathbb {R}^3$defined as

2.2 $$\begin{aligned} \Theta _h\circ z_h (x) := (x', h\theta (x')) + hx_3n_h(x'), \end{aligned}$$

where $n_h$ is a unit normal vector field relative to the mid-surface $\hat{\omega }_h:=\Theta _h \circ z_h (\bar{\omega })$, namely, the graph of the function $h\theta (\cdot )$.

For comparison to the two main reference note the following differences in notation. In [1] we have $\omega \equiv S$. In [18] we have $z_h \equiv P^h$ and consider the case $f^h \equiv h$.

#### Definition 2.4

*(Shallow shell).* With the above notation, for every $h >0$, the *shallow shell* $\hat{\Omega }_h$ with thickness *h* is defined as

$$ \hat{\Omega }_h:= \Theta _h \circ z_h(\Omega ). $$

Under the previous assumptions $\Theta _h$ is a $C^1$-diffeomorphism with the following properties:

#### Proposition 2.5

([18, Theorem 1]). Let $\Omega , \Theta _h$ and $z_h$ be as defined before. Then, there exists $h_0 = h_0(\theta ) > 0$ such that for all $h \le h_0$ the Jacobian matrix $(\nabla \Theta _h) (z_h(x))$ is invertible for all $x \in \overline{\Omega }$ and there holds

2.3 $$\begin{aligned}&(\det \nabla \Theta _h)(z_h(x)) = 1 + h^2\delta _h(z_h(x)) , \end{aligned}$$

2.4 $$\begin{aligned}&(\nabla \Theta _h)(z_h(x)) = Id - hA(x') + h^2(F_h(z_h(x)), \end{aligned}$$

2.5 $$\begin{aligned}&(\nabla \Theta _h)^{-1} (z_h(x)) = Id + hA(x') + h^2(G_h(z_h(x)), \end{aligned}$$

with

$$\begin{aligned}&\left\| {\nabla (\Theta _h)\circ z_h - Id}\right\| _{L^{\infty }(\Omega ;\mathbb {R}^{3\times 3})} \le hC,\\&\left\| {\nabla (\Theta _h)^{-1}\circ z_h - Id}\right\| _{L^{\infty }(\Omega ;\mathbb {R}^{3\times 3})} \le hC, \end{aligned}$$

where $$A(x') = \begin{pmatrix} 0 &  0 &  \partial _1\theta (x')\\ 0 &  0 &  \partial _2\theta (x')\\ -\partial _1\theta (x') &  -\partial _2\theta (x') &  0 \end{pmatrix}$$,

and $\delta _h: \overline{\Omega }_h \rightarrow \mathbb {R}$, $F_h: \overline{\Omega }_h \rightarrow \mathbb {R}^{3\times 3}$ and $G_h: \overline{\Omega }_h \rightarrow \mathbb {R}^{3\times 3}$ satisfy

$$\begin{aligned}&\underset{0<h\le h_0}{\sup }\ \ \underset{x_h \in \overline{\Omega }_h}{\max }|\delta _h(z_h(x))| \le C_0,\\&\underset{0<h\le h_0}{\sup }\ \ \underset{i,j}{\max }\ \ \underset{x_h \in \overline{\Omega }_h}{\max }|(F_h(z_h(x)))_{i,j}| \le C_0,\\&\underset{0<h\le h_0}{\sup }\ \ \underset{i,j}{\max }\ \ \underset{x_h \in \overline{\Omega }_h}{\max }|(G_h(z_h(x)))_{i,j}| \le C_0, \end{aligned}$$

for some constant $C_0 > 0$.

## Setting of the Problem and Main Results

### Setting

Consider the shallow shell with thickness *h* as defined in (2.2). We denote by $\pi _h :\hat{\Omega }_h \rightarrow \hat{\omega }_h$ the projection onto the midsurface along the normal $n_h$.

We will consider deformations $u \in W^{1,p}(\hat{\Omega }_h;\mathbb {R}^3)$ with $p > 3$. This allows us to specify pointwise the value of *u* and almost everywhere the value of $\det \nabla u$. The class of admissible deformation is made by those $u \in W^{1,p}(\hat{\Omega }_h;\mathbb {R}^3)$ such that $\det \nabla u >0$ pointwise almost everywhere and *u* is almost everywhere injective.

The magnetizations are maps $m \in W^{1,2}(\hat{\Omega }_h^u;\mathbb {S}^2)$, where $\hat{\Omega }_h^u$ is defined as in (2.1) ( recall that thanks to the fact that *u* is almost everywhere injective then by Proposition 2.2 the set $\hat{\Omega }_h^u$ is open). Additionally, we consider a map $\psi _{m}: \mathbb {R}^3 \rightarrow \mathbb {R}$ which is called the stray field potential, being the weak solution to the Maxwell equation:

3.1 $$\begin{aligned} \Delta \psi _{m} = \nabla \cdot (\chi _{\hat{\Omega }^u_h}m) \ \ \text {in} \ \mathbb {R}^3, \end{aligned}$$

where $\chi _{\hat{\Omega }^u_h}m$ extends *m* by zero to the whole space.

With this, for every $h > 0$ the energy of the magnetoelastic shallow shell $\hat{\Omega }_h$ is given for every $u \in W^{1,p}(\hat{\Omega }_h;\mathbb {R}^3)$ and $m \in W^{1,2}(\hat{\Omega }_h^u;\mathbb {S}^2)$ by

$$\begin{aligned} \mathcal {E}_h(u,m) := \frac{1}{h^\beta }\int _{\hat{\Omega }_h}W^{inc}(\nabla u, m \circ u) \ dx + \alpha \int _{\hat{\Omega }^u_h}|\nabla m|^2 \ d\xi + \frac{1}{2}\int _{\mathbb {R}^3}|\nabla \psi _m|^2 \ d\xi , \end{aligned}$$

where $\beta > 2p$ and $\alpha > 0$ and $W^{inc}: \mathbb {R}^{3\times 3}\times \mathbb {S}^2 \rightarrow [0,+\infty ]$ is called the incompressible energy density:

3.2 $$\begin{aligned} W^{inc}(F,\nu ):= \left\{ \begin{aligned} W(F,\nu ), \ \  &\det F = 1\\ +\infty , \ \  &\det F \ne 1 \end{aligned} \right. , \end{aligned}$$

where $W: \mathbb {R}^{3\times 3}\times \mathbb {S}^2 \rightarrow [0,+\infty ]$ is a continuous and nonlinear elastic energy density, which fulfills the following properties:

1. (*Frame indifference*)
  3.3 $$\begin{aligned} W(RF, R\nu ) = W(F,\nu ), \end{aligned}$$
  for every $$R \in SO(3), F \in \mathbb {R}^{3\times 3}, \nu \in \mathbb {S}^2$$,
2. (*Normalization*)
  3.4 $$\begin{aligned} W(Id, \nu ) = 0, \end{aligned}$$
  for every $$\nu \in \mathbb {S}^2$$,
3. (*Growth*) There exists a $C > 0$ such that
  3.5 $$\begin{aligned} W(F,\nu ) \ge C \text {dist}^2(F,SO(3))\vee \text {dist}^p(F,SO(3)), \end{aligned}$$
  for every $$F \in \mathbb {R}^{3\times 3}, \ \nu \in \mathbb {S}^2$$,
4. (*Regularity*) There exists a $\delta > 0$, such that for all $\nu \in \mathbb {S}^2$ the function
  3.6 $$\begin{aligned} W(\cdot ,\nu ) \ \text {is of class} \ C^2, \end{aligned}$$
  on the set $\{F \in \mathbb {R}^{3\times 3}: \text {dist}(F,SO(3)) < \delta \}$.

Frame indifference corresponds to the modelling assumption that if the observer is rotated, the energy is not affected. The normalization hypothesis incorporates the fact that the reference configuration $\hat{\Omega }_h$ is a natural state of the material with minimal energy.

By (W.2) and (W.4), we have the following second-order Taylor expansion

3.7 $$\begin{aligned} W(Id + G, \nu ) = \frac{1}{2}Q_3(G,\nu ) + \omega (G,\nu ), \end{aligned}$$

for $G \in \mathbb {R}^{3\times 3}$ with $\left| {G}\right| <\delta$ and for every $\nu \in \mathbb {S}^2$, where

$$\begin{aligned} Q_3(G,\nu ) := \mathbb {C}^\nu G:G, \end{aligned}$$

with the fourth-order tensor $\mathbb {C}^\nu \in \mathbb {R}^{3\times 3 \times 3 \times 3}$ defined by

$$\begin{aligned} \mathbb {C}^\nu := \partial _F^2W(Id, \nu ), \end{aligned}$$

and, for every $\nu \in \mathbb {S}^2$, it holds that $\omega (G,\nu ) = o(\left| {G}\right| ^2)$, as $\left| {G}\right| \rightarrow 0^+$.

By (W.2), for every $\nu \in \mathbb {S}^2$, $\mathbb {C}^\nu$ is positive definite, making the quadratic form $Q_3(.,\nu )$ convex.

In addition we assume

(W.5) The map $\nu \mapsto \mathbb {C}^\nu$ is continuous from $\mathbb {S}^2 \rightarrow \mathbb {R}^{3 \times 3 \times 3 \times 3}$, and
  $$\begin{aligned} \overline{\omega }(t) := \sup \big \{ \frac{\omega (G,\nu )}{\left| {G}\right| ^2} \ : \ G \in \mathbb {R}^{3 \times 3}, \left| {G}\right| \le t, \nu \in \mathbb {S}^2 \big \} = o(t^2), \ \text {as} \ t \rightarrow 0^+. \end{aligned}$$

### Fixing the Domain

As customary in dimension reduction problems, we perform a change of variables to rewrite $\mathcal {E}_h(u,m)$ as an integral functional on the fixed domain $\Omega$ and a fixed deformed domain which is the counterpart to $\hat{\Omega }_h^u$. Using the fact that for for $h > 0$ small, $\Theta _h \circ z_h$ is a $C^1-$Diffeomorphism, we first write the elastic energy functional in terms of the map $u\circ \Theta _h \circ z_h \in W^{1,p}(\Omega ;\mathbb {R}^3)$:

$$\begin{aligned} \mathcal {E}_h(u,m)&= \frac{1}{h^\beta }\int _{\Theta _h(z_h(\Omega ))}W^{inc}(\nabla u, m \circ u) \ dx + \alpha \int _{\hat{\Omega }^u_h}|\nabla m|^2 \ d\xi + \frac{1}{2}\int _{\mathbb {R}^3}|\nabla \psi _m|^2 \ d\xi \\&= \frac{1}{h^\beta }\int _{\Omega }W^{inc}((\nabla u) \circ \Theta _h \circ z_h, m \circ u \circ \Theta _h \circ z_h)\left| {\det \nabla (\Theta _h \circ z_h)}\right| \ dx\\&\quad + \alpha \int _{\hat{\Omega }^u_h}|\nabla m|^2 \ d\xi + \frac{1}{2}\int _{\mathbb {R}^3}|\nabla \psi _m|^2 \ d\xi . \end{aligned}$$

By the chain rule, there holds

$$\begin{aligned} \left| {\det \nabla (\Theta _h \circ z_h) (x)}\right| = \left| {\det ((\nabla \Theta _h) \circ z_h(x))\det (\nabla z_h(x))}\right| = h\left| {\det ((\nabla \Theta _h) \circ z_h(x))}\right| . \end{aligned}$$

We therefore obtain

$$\begin{aligned} h^{-1}\mathcal {E}_h(u,m)&= \frac{1}{h^\beta }\int _{\Omega }W((\nabla u) \circ \Theta _h \circ z_h, m \circ u \circ \Theta _h \circ z_h) \left| {\det ((\nabla \Theta _h) \circ z_h)}\right| \ dx\\&\quad + \frac{\alpha }{h}\int _{\hat{\Omega }^u_h}|\nabla m|^2 \ d\xi + \frac{1}{2h}\int _{\mathbb {R}^3}|\nabla \psi _m|^2 \ d\xi . \end{aligned}$$

Notice, that again by the chain rule the following identity holds true:

$$\begin{aligned} (\nabla u)\circ \Theta _h \circ z_h = \nabla _h(u\circ \Theta _h\circ z_h)((\nabla \Theta _h)^{-1} \circ z_h). \end{aligned}$$

We set

3.8 $$\begin{aligned}&\kappa _h := \left| {\det ((\nabla \Theta _h) \circ z_h(x))}\right| , \end{aligned}$$

3.9 $$\begin{aligned}&M_h := ((\nabla \Theta _h)^{-1} \circ z_h), \end{aligned}$$

3.10 $$\begin{aligned}&y := u \circ \Theta _h \circ z_{h|_\Omega }, \end{aligned}$$

for every $h > 0$. With this he rescaled magnetoelastic energy functional $E_h = h^{-1}\mathcal {E}_h$ is given by

$$\begin{aligned} E_h(y, m) := \frac{1}{h^{\beta }}\int _{\Omega } W^{inc}(\nabla _h y M_h, m \circ y) \ \kappa _h \ dx + \frac{\alpha }{h}\int _{\Omega ^y}|\nabla m|^2 \ d\xi + \frac{1}{2h}\int _{\mathbb {R}^3}|\nabla \psi _m|^2 \ d\xi , \end{aligned}$$

for every pair of admissible states (*y*, *m*).

#### Definition 3.1

*(Admissible states).* We call a deformation *y* admissible if it belongs to the set

$$\begin{aligned} \mathcal {Y} := \big \{ y \in W^{1,p}(\Omega ,\mathbb {R}^3) \ : \ \det \nabla y > 0 \ \text {a.e. in} \ \Omega , \ y \ \text {a.e. injective in} \ \Omega \big \}. \end{aligned}$$

The associated admissible magnetizations are maps $m \in W^{1,2}(\Omega ^y;\mathbb {S})$, with $\Omega ^y$ as in (2.1). Eventually, the set of admissible states $\mathcal {Q}$ is given by

$$\begin{aligned} \mathcal {Q} := \big \{ (y,m) \ | \ y \in \mathcal {Y}, \ m \in W^{1,2}(\Omega ^y;\mathbb {S}) \big \}. \end{aligned}$$

We split the energy into three parts: the elastic energy $E^{el}_h$, the exchange energy $E^{exc}_h$ and the magnetostatic energy $E^{mag}_h$, given by

$$\begin{aligned}&E^{el}_h := \frac{1}{h^{\beta }}\int _{\Omega } W^{inc}(\nabla _h y M_h, m \circ y) \ \kappa _h \ dx\\&E^{exc}_h := \frac{\alpha }{h}\int _{\Omega ^y}|\nabla m|^2 \ d\xi \\&E^{mag}_h := \frac{1}{2h}\int _{\mathbb {R}^3}|\nabla \psi _m|^2 \ d\xi . \end{aligned}$$

With the following Lemma, we collect some properties of the quantities introduced above relative to the geometry of the shallow shell that are uselful in upcoming proofs.

#### Lemma 3.2

For the maps $\kappa _h$ and $M_h$ defined in (3.8) and (3.9) and $h > 0$ sufficiently small, there holds

3.11 $$\begin{aligned}&\kappa _h \le C_1, \end{aligned}$$

3.12 $$\begin{aligned}&\kappa _h \ge C_2, \end{aligned}$$

3.13 $$\begin{aligned}&\left\| {M_h}\right\| _{L^\infty (\Omega ;\mathbb {R}^{3\times 3})} \le C_1, \end{aligned}$$

3.14 $$\begin{aligned}&\left\| {M_h}\right\| _{L^\infty (\Omega ;\mathbb {R}^{3\times 3})} \ge C_2, \end{aligned}$$

3.15 $$\begin{aligned}&\left\| {M_h \xi }\right\| _{L^2(\mathbb {R}^3;\mathbb {R}^{3\times 3})} \ge C \ \left\| {\xi }\right\| _{L^2(\mathbb {R}^3;\mathbb {R}^{3\times 3})}, \end{aligned}$$

for all $\xi \in \mathbb {R}^3$, where $C_1, C_2, C > 0$. Moreover, as $h \rightarrow 0^+$ for all $1 \le r \le \infty$, there holds

3.16 $$\begin{aligned}&\kappa _h \rightarrow 1 \ \ \text {in} \ L^r(\Omega ), \end{aligned}$$

3.17 $$\begin{aligned}&M_h \rightarrow Id \ \ \text {in} \ L^r(\Omega ; \mathbb {R}^{3 \times 3}) \end{aligned}$$

#### Proof

This Lemma is a direct consequence of Proposition 2.5. By (2.3), it follows that

$$\begin{aligned} \kappa _h = \left| {1 + h^2\delta _h \circ z_h}\right| , \end{aligned}$$

and also

$$\begin{aligned} \kappa _h&\le 1 + h^2C_0,\\ \kappa _h&\ge 1 - h^2C_0. \end{aligned}$$

Inequalities (3.11) and (3.12) follow from this observation if $h > 0$ is sufficiently small. Property (3.13) holds because of (2.5). By the inverse triangle inequality, there holds

$$\begin{aligned} \left\| {M_h}\right\| _{L^\infty (\Omega ;\mathbb {R}^{3\times 3})}&= \left\| {Id + h(A \circ \pi ) + h^2(G_h \circ z_h)}\right\| _{L^\infty (\Omega ;\mathbb {R}^{3\times 3})}\\&\ge \left\| {Id + h(A \circ \pi )}\right\| _{L^\infty (\Omega ;\mathbb {R}^{3\times 3})} - h^2 \left\| {G_h \circ z_h}\right\| _{L^\infty (\Omega ;\mathbb {R}^{3\times 3})}\\&\ge \left\| {Id}\right\| _{L^\infty (\Omega ;\mathbb {R}^{3\times 3})} - h \left\| {A \circ \pi }\right\| _{L^\infty (\Omega ;\mathbb {R}^{3\times 3})} - h^2 \left\| {G_h \circ z_h}\right\| _{L^\infty (\Omega ;\mathbb {R}^{3\times 3})}\\&\ge 1 - hC - h^2C_0. \end{aligned}$$

For *h* small enough (3.14) follows. By an analogous argument (3.15) is shown.

Eventually, (3.16) and (3.17) follow from (2.3) and (2.5). $\square$

### Main Results

First we introduce the reduced energy functional. Analogously to [1], for every $H \in \mathbb {R}^{2\times 2}$ and $\nu \in \mathbb {S}^2$ we set

$$\begin{aligned} \begin{aligned} Q_2^{inc}(H, \nu ) := \min \Bigg \{&Q_3\bigg (\left( \begin{array}{c|c} H &  0'\\ \hline (0')^T &  0 \end{array}\right) + c \otimes e_3 + e_3 \otimes c, \nu \bigg ) : c \in \mathbb {R}^3, \\&\text {tr}\bigg (\left( \begin{array}{c|c} H &  0'\\ \hline (0')^T &  0 \end{array}\right) + c \otimes e_3 + e_3 \otimes c\bigg ) = 0\Bigg \}. \end{aligned} \end{aligned}$$

With this, for every triple $(u,v,\lambda ) \in W^{1,2}(\omega ;\mathbb {R}^2) \times W^{2,2}(\omega ) \times W^{1,2}(\omega ;\mathbb {S}^2)$ we define the reduced energy as

$$\begin{aligned} \begin{aligned} E(u,v,\lambda )&:= \frac{1}{2}\int _\omega Q_2^{inc}(\text {sym}(\nabla ' u + \nabla 'v \otimes \nabla '\theta ), \lambda ) \ dx'\\&\quad + \ \frac{1}{24}\int _\omega Q_2^{inc}((\nabla ')^2v, \lambda ) \ dx' + \alpha \int _\omega \left| {\nabla ' \lambda }\right| ^2 \ dx' + \frac{1}{2}\int _\omega \left| {(\lambda )^3}\right| ^2 \ dx'. \end{aligned} \end{aligned}$$

We again split the above energy functional into three parts as follows

$$\begin{aligned}&E^{el}(u,v,\lambda ) = \frac{1}{2}\int _\omega Q_2^{inc}(\text {sym}(\nabla ' u + \nabla 'v \otimes \nabla '\theta ), \lambda ) \ dx' + \frac{1}{24}\int _\omega Q_2^{inc}((\nabla ')^2v, \lambda ) \ dx'\\&E^{exc}(\lambda ) = \alpha \int _\omega \left| {\nabla ' \lambda }\right| ^2 \ dx'\\&E^{mag}(\lambda ) = \frac{1}{2}\int _\omega \left| {(\lambda )^3}\right| ^2 \ dx'. \end{aligned}$$

Lastly, we introduce in-plane and out-of-plane displacements.

#### Definition 3.3

*(In-plane and out-of-plane displacement).*

Let $y_h \in W^{1,p}(\Omega ;\mathbb {R}^3)$ be a deformation. The associated in-plane displacement $U_h^{y_h}: \omega \rightarrow \mathbb {R}^2$ and out-of-plane displacement $V_h^{y_h}: \omega \rightarrow \mathbb {R}$ are given by

3.18 $$\begin{aligned}&U_h^{y_h}(x') := \frac{1}{h^{\frac{\beta }{2}}}\int _{-\frac{1}{2}}^{\frac{1}{2}}\big (y_h'(x',x_3) - x'\big ) \ dx_3, \end{aligned}$$

3.19 $$\begin{aligned}&V_h^{y_h}(x') := \frac{1}{h^{\frac{\beta }{2}-1}}\int _{-\frac{1}{2}}^{\frac{1}{2}}\big ((y_h)^3(x',x_3) - h\theta (x')\big ) \ dx_3. \end{aligned}$$

We are now in position to state the two main contributions of this paper.

#### Theorem 3.4

(Compactness and lower bound).

Assume $p > 3$ and $\beta > 2p$ and that the elastic energy density *W* satisfies (W.1)-3. Let $((y_h,m_h))_h \subseteq \mathcal {Q}$ be a sequence of admissible states which satisfies $E_h(y_h,m_h) \le C$ for every $h>0$. Then, there exists a sequence of rotations $(Q_h)_h \subseteq SO(3)$ and a sequence of translation vectors $(c_h)_h \subseteq \mathbb {R}^3$, such that by setting $\tilde{y}_h:= T_h \circ y_h$ and $\tilde{m}_h:= Q_h^Tm_h \circ T_h^{-1}$, where $T_h: \mathbb {R}^3 \rightarrow \mathbb {R}^3$ is the rigid motion defined by $T_h(\xi ):= Q_h^T\xi - c_h$ for every $\xi \in \mathbb {R}^3$, we have, as $h \rightarrow 0^+$:

$$\begin{aligned}&U_h^{\tilde{y}_h} \rightharpoonup u \ \ \text {in} \ W^{1,2}(\omega ;\mathbb {R}^3) \ \ \text {for some} \ u \in W^{1,2}(\omega ;\mathbb {R}^3);\\&V_h^{\tilde{y}_h} \rightarrow v \ \ \text {in} \ W^{1,2}(\omega ) \ \ \text {for some} \ v \in W^{2,2}(\omega );\\&\tilde{m}_h \circ \tilde{y}_h \rightarrow \lambda \ \ \text {in} \ L^r(\Omega ;\mathbb {R}^3) \ \ \text {for every} \ 1 \le r < \infty , \ \text {for some} \ \lambda \in W^{1,2}(\omega ;\mathbb {S}^2). \end{aligned}$$

Moreover, the following inequality holds:

$$\begin{aligned} E(u,v,\lambda ) \le \liminf _{h\rightarrow 0^+}E_h(y_h,m_h). \end{aligned}$$

#### Theorem 3.5

(Recovery sequence). Assume $p > 3$ and $\beta > 2p$ and that the elastic energy density *W* satisfies (W.1)-3. Then, for every $(u,v,\lambda ) \in W^{1,2}(\omega ;\mathbb {R}^2) \times W^{2,2}(\omega ) \times W^{1,2}(\omega ;\mathbb {S}^2)$, there exists a sequence of admissible states $((y_h,m_h))_h \subseteq \mathcal {Q}$ such that, as $h \rightarrow 0^+$:

$$\begin{aligned}&u_h := U_h^{y_h} \rightarrow u \ \ \text {in} \ W^{1,2}(\omega ;\mathbb {R}^3);\\&v_h := V_h^{y_h} \rightarrow v \ \ \text {in} \ W^{1,2}(\omega ));\\&m_h \circ y_h \rightarrow \lambda \ \ \text {in} \ L^r(\Omega ;\mathbb {R}^3) \ \ \text {for every} \ 1 \le r < \infty . \end{aligned}$$

Moreover, the following equality holds:

$$\begin{aligned} \lim _{h\rightarrow 0^+}E_h(y_h,m_h) = E(u,v,\lambda ). \end{aligned}$$

Analogously to [1, Section 3.4], we can state the two main results from Theorem 3.4 and 3.5 in terms of $\Gamma$-convergence. For this, we first introduce the functional $\mathcal {M}_h(y,m): \mathbb {R}^3 \rightarrow \mathbb {R}^3$ by setting

$$\begin{aligned} \mathcal {M}_h(y,m) := (\chi _{\Omega ^{y}}m)\circ \Theta _h\circ z_h, \end{aligned}$$

for $h > 0$ and admissible states $(y,m) \in \mathcal {Q}$.

Recalling (3.18) and (3.19), we introduce the functionals

$$\begin{aligned} \mathcal {I}_h, \mathcal {I}: W^{1,p}(\Omega ;\mathbb {R}^3) \times W^{1,2}(\omega ;\mathbb {R}^2)\times W^{1,2}(\omega )\times L^2(\mathbb {R}^3;\mathbb {R}^3) \rightarrow \mathbb {R}^3\cup \{+\infty \}, \end{aligned}$$

given by

$$\begin{aligned} \mathcal {I}_h(y,u,v,\mu ) := {\left\{ \begin{array}{ll} E_h(y,m) &  \text {if} \ u = U_h^{y}, v = V_h^y \ \text {and} \ \mu = \mathcal {M}_h(y,m)\\ &  \text {for some} \ m \in W^{1,2}(\Omega ^y;\mathbb {S}^2), \\ +\infty &  \text {otherwise} \end{array}\right. } \end{aligned}$$

and

$$\begin{aligned} \mathcal {I}(y,u,v,\mu ) := {\left\{ \begin{array}{ll} E(u,v,\lambda ) &  \text {if} \ y = \Theta _0 \circ z_0, v \in W^{2,2}(\omega ) \ \text {and} \ \mu = \chi _\Omega \lambda \\ &  \text {for some} \ \lambda \in W^{1,2}(\Omega ^y;\mathbb {S}^2), \\ +\infty &  \text {otherwise}. \end{array}\right. } \end{aligned}$$

Similarly to [1, Corollary 3.1], we obtain the following result:

#### Corollary 3.6

($\Gamma$-convergence). The functionals $(\mathcal {I}_h)$ $\Gamma$-converge to $\mathcal {I}$ with respect to the strong product topology, as $h \rightarrow 0^+$. Namely, the following holds:

(i) (Liminf inequality) for every sequence $((y_h,u_h,v_h,\mu _h))_h \subseteq W^{1,p}(\Omega ;\mathbb {R}^3) \times W^{1,2}(\omega ;\mathbb {R}^2)\times W^{1,2}(\omega )\times L^2(\mathbb {R}^3;\mathbb {R}^3)$ such that $(y_h,u_h,v_h,\mu _h) \rightarrow (y,u,v,\mu )$ in $W^{1,p}(\Omega ;\mathbb {R}^3) \times W^{1,2}(\omega ;\mathbb {R}^2)\times W^{1,2}(\omega )\times L^2(\mathbb {R}^3;\mathbb {R}^3)$ for some $(y,u,v,\mu ) \in W^{1,p}(\Omega ;\mathbb {R}^3) \times W^{1,2}(\omega ;\mathbb {R}^2)\times W^{1,2}(\omega )\times L^2(\mathbb {R}^3;\mathbb {R}^3)$, we have
  $$\begin{aligned} \mathcal {I}(y,u,v,\mu ) \le \underset{h \rightarrow 0^+}{\liminf }\ \ \mathcal {I}_h(y_h,u_h,v_h,\mu _h), \end{aligned}$$
(ii) (Recovery sequence) for every $(y, u, v, \mu ) \in W^{1,p}(\Omega ;\mathbb {R}^3) \times W^{1,2}(\omega ;\mathbb {R}^2)\times W^{1,2}(\omega )\times L^2(\mathbb {R}^3;\mathbb {R}^3)$ there exists a sequence $((y_h,u_h,v_h,\mu _h))_h \subseteq W^{1,p}(\Omega ;\mathbb {R}^3) \times W^{1,2}(\omega ;\mathbb {R}^2)\times W^{1,2}(\omega )\times L^2(\mathbb {R}^3;\mathbb {R}^3)$ such that $(y_h,u_h,v_h,\mu _h) \rightarrow (y,u,v,\mu )$ in $W^{1,p}(\Omega ;\mathbb {R}^3) \times W^{1,2}(\omega ;\mathbb {R}^2)\times W^{1,2}(\omega )\times L^2(\mathbb {R}^3;\mathbb {R}^3)$ and we have
  $$\begin{aligned} \mathcal {I}(y, u, v, \mu ) = \underset{h \rightarrow 0^+}{\lim }\ \ \mathcal {I}_h(y_h, u_h, v_h, \mu _h). \end{aligned}$$

The same result holds for the weak product topology instead of the strong one.

#### Proof

With Theorem 3.4 and Theorem 3.5 we obtain the result by a simple adaptation of [1, Corollary 3.1] using Lemma 3.2 to obtain bounds for the shallow shell map $\Theta _h\circ z_h$. $\square$

Corollary 3.6 offers less information than the two main results, as the sequence of functionals $(\mathcal {I}_h)$ are not coercive. Consequently, the Fundamental Theorem of $\Gamma$-convergence is not applicable and the convergence of minimizers cannot be deduced.

## Approximation by Rotations and Compactness

### Approximation by Rotations

This section is dedicated to the first crucial step for the proof of Theorem 3.4. The following approximation result for magnetoelastic shallow shells hinges on the rigidity estimate stated in Theorem 2.3. Before stating it, we observe that the rotation *R* appearing in Theorem 2.3 does not depend on the exponent *p* (see [1, Remark 2.3]). This immediately implies that for every $u \in W^{1,p}(\Omega ;\mathbb {R}^3)$, we find a rotation $R \in SO(3)$ such that

4.1 $$\begin{aligned} \int _\Omega \left| {\nabla u(x) - R}\right| ^q dx \le C(\Omega , q) K(u,\Omega ) , \end{aligned}$$

for $q \in \{2,p\}$, whenever we set

$$\begin{aligned} K(u,\Omega ) := \int _\Omega \text {dist}^2(\nabla u, SO(3)) \vee \text {dist}^p(\nabla u, SO(3))dx. \end{aligned}$$

Suppose that our energy sequence is equi-bounded, meaning that

$$\begin{aligned} E_h(y_h, m_h) \le C, \ \ \ \forall \ h > 0. \end{aligned}$$

Then, by the growth condition (W.3) there holds

4.2 $$\begin{aligned} K_h(y_h,\Omega ) \le h^{\beta }E^{el}_h(y_h,m_h) \le Ch^{\beta }. \end{aligned}$$

In the next theorem we extend the approximation by rotations result obtained in [1, Lemma 4.1] to the setting of magnetoelastic shallow shells.

#### Proposition 4.1

(Approximation by rotations). Let $u_h \in W^{1,p}(\hat{\Omega }_h;\mathbb {R}^3)$ and assume that $h^{-p}K(u_h,\hat{\Omega }_h)$ is sufficiently small, for $h \ll 1$.

Then, there exist matrix fields $R_h \in W^{1,p}(\hat{\omega }_h;\mathbb {R}^{3\times 3})$ such that $R_h(\pi _h(Z)) \in SO(3)$ for all $Z \in \hat{\Omega }_h$, and matrices $Q_h \in SO(3)$ with the following properties:

$$\begin{aligned}&(i) \ \left\| {\nabla u_h - R_h\pi _h}\right\| _{L^q(\hat{\Omega }_h;\mathbb {R}^{3 \times 3})}^q \le CK(u_h,\hat{\Omega }_h),\\&(ii) \ \left\| { \hat{\nabla } R_h }\right\| _{L^q(\hat{\omega }_h;\mathbb {R}^{3 \times 3})}^q \le Ch^{-q-1}K(u_h,\hat{\Omega }_h),\\&(iii) \ \left\| {R_h - Q_h}\right\| _{L^q(\hat{\omega }_h;\mathbb {R}^{3 \times 3})}^q \le Ch^{-q-1}K(u_h,\hat{\Omega }_h), \end{aligned}$$

where the constant *C* is independent of $u_h$ and *h*.

#### Proof

**Step 1** (Applying the rigidity estimate locally):

Consider a ball $B_h(X) \subseteq \mathbb {R}^3$ with radius *h* centered at $X \in \hat{\omega }_h$ and set

$$\begin{aligned} \begin{aligned} D_{X,h} := B_h(X) \cap \hat{\omega }_h\\ B_{X,h} := \pi ^{-1}_h(D_{X,h}) \cap \hat{\Omega }_h. \end{aligned} \end{aligned}$$

The cylinders $B_{X,h}$ are open and bounded domains with Lipschitz boundaries. This allows us to apply the Rigidity Estimate (4.1) to find rotations $R_{X,h}$ such that

4.3 $$\begin{aligned} \int _{B_{X,h}} \left| {\nabla u_h(y) - R_{X,h}}\right| ^q dy \le C K(u_h,B_{X,h}). \end{aligned}$$

By the fact that $\Theta _h \circ z_h$ is a $C^1$-Diffeomorphism, we know that $\hat{\Omega }_h$ is bi-Lipschitz equivalent to $\Omega _h$. As a consequence, all cylinders $B_{X,h}$ are bi-Lipschitz equivalent to each other for every sufficiently small $h >0$. Hence we can apply Theorem 2.3 to infer that the constant *C* in the previous estimate can be chosen independently from *h* and *x*.

**Step 2** (Cut-off function):

Take a function $\rho \in \mathcal {C}_c^\infty (\mathbb {R};[0,1])$ with supp$(\rho ) \subseteq (-\frac{1}{2},\frac{1}{2})$, and $\int _{\mathbb {R}}\rho = 1$ which is equal to 1 in a small neighbourhood of 0. For $X \in \hat{\omega }_h$ define the function $\eta _X: \hat{\omega }_h \rightarrow \mathbb {R}$ by setting

$$\begin{aligned} \eta _X(Z) = \frac{\rho (\left| {\pi _h Z - X}\right| /h)}{\int _{\hat{\Omega }_h}\rho (\left| {\pi _h Y - X}\right| /h) \ dY}. \end{aligned}$$

Then, $\eta _X$ satisfies the following:

4.4 $$\begin{aligned}&\text {For} \ Z \notin B_{X,h}, \ \ \eta _X(Z) = 0, \end{aligned}$$

4.5 $$\begin{aligned}&\int _{\hat{\Omega }_h}\eta _X(Z) \ dZ = 1, \end{aligned}$$

4.6 $$\begin{aligned}&\left\| {\eta _X}\right\| _{L^\infty (\hat{\Omega }_h)} \le Ch^{-3}, \end{aligned}$$

4.7 $$\begin{aligned}&\left\| {\nabla \eta _X}\right\| _{L^\infty (\hat{\Omega }_h;\mathbb {R}^3)} \le Ch^{-4}. \end{aligned}$$

Property (4.4) follows from the fact that $Z \notin B_{X,h}$ implies $\left| {\pi _h Z - X}\right| /h > 1$ and consequently there holds $\rho (\left| {\pi _h Z - X}\right| /h) = 0$. Condition (4.6) can be shown by using $\left\| {\rho }\right\| _{L^\infty (\mathbb {R})} = 1$ and applying the change of variables $\tilde{Y} = (Y - X)/h$. The argument for (4.7) is analogous with the addition of using $\left\| {\nabla _x\rho }\right\| _{L^\infty (\mathbb {R})} \le Ch^{-1}$.

**Step 3** (Global estimate):

Consider the matrix field $\tilde{R} \in W^{1,p}(\hat{\omega }_h;\mathbb {R}^{3\times 3})$ given by

$$\begin{aligned} \tilde{R}(X) = \int _{\hat{\Omega }_h}\eta _X(Z)\nabla u(Z) \ dZ. \end{aligned}$$

Using (4.4), Hölder inequality together with (4.5) and the local rigidity estimate (4.3), we obtain:

4.8 $$\begin{aligned} \begin{aligned} \left| {\tilde{R}(X) - R_{X,h}}\right| ^q&= \left| {\int _{B_{X,h}}\eta _X(Z)\nabla u(Z) \ dz - R_{X,h}\int _{B_{X,h}}\eta _X(Z) \ dZ }\right| ^q\\&= \left| {\int _{B_{X,h}}\eta _X(Z)\big (\nabla u(Z) - R_{X,h}\big ) \ dZ}\right| ^q\\&\le \left\| {\eta _X}\right\| _{L^\infty (\hat{\Omega }_h)}^q\mathcal {L}^3(B_{X,h})^{q-1} \int _{B_{X,h}}\left| {\nabla u (Z) - R_{X,h}}\right| ^q \ dz\\&\le Ch^{-3} K(u_h,B_{X,h}). \end{aligned} \end{aligned}$$

Similarily, using in addition (4.7), we get the following estimate:

4.9 $$\begin{aligned} \begin{aligned} \left| {\hat{\nabla } \tilde{R}(X)}\right| ^q&= \left| {\nabla _X\bigg (\int _{\hat{\Omega }_h}\eta _X(Z)\nabla u(Z) \ dZ\bigg )}\right| ^q\\&= \left| {\int _{B_{X,h}}(\nabla _X\eta _X (Z)) \big (\nabla u(Z) - R_{X,h}\big ) \ dZ}\right| ^q\\&\le \left\| {\nabla _X\eta _X}\right\| ^q_{L^\infty (\hat{\Omega }_h)}\mathcal {L}^3(B_{X,h})^{q-1}\int _{B_{X,h}}\left| {\nabla u(Z) - R_{X,h}}\right| ^q \ dZ\\&\le Ch^{-q-3}K(u_h,B_{X,h}). \end{aligned} \end{aligned}$$

For any $\tilde{X} \in B_{X,h} \cap \hat{\omega }_h$, using the Fundamental Theorem of Calculus, by setting $\gamma _h(t):= \Theta _h(x' + t(\tilde{x}' - x'),0)$ for $t \in [0,1]$, we infer

$$\begin{aligned} \left| {\tilde{R}(\tilde{X}) - \tilde{R}(X)}\right|&= \left| {\int _0^1 \frac{d}{dt} \tilde{R}(\gamma _h(t)) \ dt}\right| \\&= \left| {\int _0^1 \nabla \tilde{R}(\gamma _h(t)) \cdot \dot{\gamma }_h(t) \ dt}\right| . \end{aligned}$$

By the Mean Value Theorem, we find $\tau \in [0,1]$ such that

$$\begin{aligned} \left| {\int _0^1 \nabla \tilde{R}(\gamma _h(t)) \cdot \dot{\gamma }_h(t) \ dt}\right| \le \text {length}(\gamma _h) \ \left| {\nabla \tilde{R}(\gamma _h(\tau ))}\right| . \end{aligned}$$

Since $\text {length}(\gamma _h) \le |x'-\tilde{x}'| + h \text {Lip}(\theta ) \le Ch$, for some $C > 0$, writing $Y_h:= \gamma _h(\tau )$ and using (4.9), we deduce

4.10 $$\begin{aligned} \begin{aligned} \left| {\tilde{R}(\tilde{X}) - \tilde{R}(X)}\right| ^q&\le C h^q \left| {\hat{\nabla }\tilde{R}(Y_h)}\right| ^q\\&\le C h^{-3}K(u_h, B_{Y_h,h})\\&\le C h^{-3}K(u_h, B_{X,2h}), \end{aligned} \end{aligned}$$

Combining (4.3), (4.8) and (4.10) results in

4.11 $$\begin{aligned} \begin{aligned}&\int _{B_{X,h}}\left| {\nabla u(Z) - \tilde{R}(\pi _h(Z))}\right| ^q \ dZ\\&\le \int _{B_{X,h}}\bigg (\left| {\nabla u(Z) - R_{X,h}}\right| + \left| {\tilde{R}(X) - R_{X,h}}\right| + \left| {\tilde{R}(\pi _h(Z)) - \tilde{R}(X)}\right| \bigg )^q \ dZ\\&\le CK(u_h, B_{X,2h}). \end{aligned} \end{aligned}$$

**Step 4** (Covering):

Consider a square $Q = [-a,a]^2 \subseteq \mathbb {R}^3$, for some $a > 0$ such that $\omega \subseteq Q$ as well as the equidistant partition $(\frac{n}{2^i})_{n=0}^{2^i} \subseteq [0,1]$ with distance $\frac{1}{2^i}$ for some $i \in \mathbb {N}$. Using the affine transformation $\varphi : [0,1] \rightarrow [-a,a]$, defined as

$$\begin{aligned} \varphi (x) := -a(1-2x), \end{aligned}$$

with $\{\varphi (\frac{n}{2^i})\}_n$ we get an equidistant partition of $[-a,a]$ with distance $\frac{2a}{2^i}$. With this partition we can define a grid of equidistant points $(x'_n)_{n=0}^{N_i}$ with distance $\frac{2a}{2^i}$ to their neighbors on *Q*. Define now $D_{r_i}(x'_n)$ which are discs with radius $r_i = \frac{4a}{2^i}$ around $x'_n$. By construction it holds that $\omega \subseteq \bigcup _{n = 0}^{N_i}D_{r_i}(x'_n)$. Now suppose that $\frac{2a}{2^i} \sim h$, or to be more precise $i:= \bigl \lceil \log _2(\frac{2a}{h})\bigr \rceil$. Then, for all points $x' \in \omega$ the covering number of the discs is independent of *h*. As the map $\Theta _h \circ z_h$ is bijective this implies that the covering of $\hat{\omega }_h$ by $\Theta _h\circ z_h (D_{r_i}(x'_n))$ is again independent of *h*. As the map $\pi _h$ is well defined, the independence is preserved if we consider $B_{X_n,h}:= \pi _h^{-1}(\Theta _h\circ z_h (D_{r_i}(x'_n))) \cap \hat{\Omega }_h$.

This implies the existence of a uniform constant *C* which does not depend on *h* (or *i*) such that by summing up the local rigidity estimates from (4.11), we get

$$\begin{aligned} \int _{\hat{\Omega }_h}\left| {\nabla u_h - \tilde{R}\pi _h}\right| ^q \ dX \le C\sum _{n=1}^{N_i}K(u_h, B_{X_n,2h}). \end{aligned}$$

By definition we have $\bigcup _{n = 1}^{N_i}B_{X_n,2h} = \hat{\Omega }_h$ and therefore

$$\begin{aligned} \sum _{n=1}^{N_i}K(u_h, B_{X_n,2h}) = K(u_h,\hat{\Omega }_h), \end{aligned}$$

such that

4.12 $$\begin{aligned} \int _{\hat{\Omega }_h}\left| {\nabla u_h - \tilde{R}\pi _h}\right| ^q \le C K(u_h, \hat{\Omega }_h). \end{aligned}$$

Integrating $\left| {\nabla \tilde{R}}\right| ^q$ over $\pi _h(B_{X_n,h})$ and using (4.9) results in

$$\begin{aligned} \int _{\pi _h(B_{X_n,h})}\left| {\hat{\nabla } \tilde{R}}\right| ^q \ dX&\le \mathcal {L}^2(\pi _h(B_{X_n,h})) \max _{Y \in \pi _h(B_{X_n,h})}\left| {\hat{\nabla } \tilde{R}(Y)}\right| ^q\\&\le Ch^{2}h^{-q-3}\max _{Y \in \pi _h(B_{X_n,h})}\\&\quad \int _{B_{Y,h}}\text {dist}^p(\nabla u_h, SO(3)) \vee \text {dist}^2(\nabla u_h, SO(3)) \ dX\\&\le Ch^{-q-1}\int _{B_{X_n,2h}}\text {dist}^p(\nabla u_h, SO(3)) \vee \text {dist}^2(\nabla u_h, SO(3)) \ dX\\&= Ch^{-q-1}K(u_h,B_{X_n,2h}). \end{aligned}$$

Using $\bigcup _{n = 1}^{N_i}\pi _h(B_{X_n,h}) = \hat{\omega }_h$, we deduce (ii).

**Step 5** (Showing (i)):

From (4.8) it follows that

$$\begin{aligned} \text {dist}^q(\tilde{R}(X), SO(3)) \le Ch^{-3}K(u_h(X), \hat{\Omega }_h). \end{aligned}$$

By (4.2) we know that $h^{-3}K(u, \hat{\Omega }_h)$ is sufficiently small, which implies that the orthogonal projection of $\tilde{R}$ onto *SO*(3)

$$\begin{aligned} R := \Pi _{SO(3)}\tilde{R}. \end{aligned}$$

is well defined.

Since $\tilde{R} \in W^{1,p}(\hat{\omega }_h;\mathbb {R}^{3\times 3})$ and $\Pi _{SO(3)}$ is Lipschitz, it follows that $R \in W^{1,p}(\hat{\omega }_h;SO(3))$.

As a next step, we show

4.13 $$\begin{aligned} \int _{\hat{\Omega }_h}\text {dist}^q(\tilde{R}\circ \pi _h, SO(3)) \ dX \le \int _{\hat{\Omega }_h}\text {dist}^q(\nabla u_h, SO(3)) \ dX. \end{aligned}$$

Using the fact that $\bigcup _{n = 1}^{N_i}B_{X_n,2h} = \hat{\Omega }_h$ and

$$\begin{aligned} \left| {\tilde{R}(\pi _h(X)) - \Pi _{SO(3)}(\tilde{R}(\pi _h(X)))}\right| ^q \le \left| {\tilde{R}(\pi _h(X)) - R_{x_i,h}}\right| ^q, \end{aligned}$$

we get

$$\begin{aligned}&\ \ \int _{\hat{\Omega }_h}\text {dist}^q(\tilde{R} (\pi _h(X)), SO(3)) \ dX = \int _{\hat{\Omega }_h}\left| {\tilde{R}(\pi _h(X)) - \Pi _{SO(3)}\tilde{R}(\pi _h(X))}\right| ^q \ dX\\&\le C \sum _{n = 1}^{N_i} \int _{B_{X_n,h}}\left| {\tilde{R}(\pi _h(X)) - R_{X_n,h}}\right| ^q \ dX\\&= C \sum _{n = 1}^{N_i} \int _{B_{X_n,h}}\left| {\int _{\hat{\Omega }_h}\eta _X(Z)\nabla u_h(Z) \ dZ - R_{X_n,h}}\right| ^q \ dX\\&= C \sum _{n = 1}^{N_i} \int _{B_{X_n,h}}\left| {\int _{\hat{\Omega }_h}(\nabla u_h(Z) - R_{X_n,h})\eta _X(Z) \ dZ}\right| ^q \ dX. \end{aligned}$$

We now use the fact that $\eta _X(Z) = 0$ for $Z \notin B_{X,h}$ and Jensen’s inequality for the function $f \mapsto \left| {f}\right| ^q$. Note also that since $X \in B_{X_n,h}$ it follows that $B_{X,h} \subseteq B_{X_n,2h}$. Together this yields

$$\begin{aligned}&\sum _{n = 1}^{N_i} \int _{B_{X_n,h}}\left| {\int _{\hat{\Omega }_h}(\nabla u_h(Z) - R_{X_n,h})\eta _X(Z) \ dZ}\right| ^q \ dX\\&\le \sum _{n = 1}^{N_i} \int _{B_{X_n,h}}\bigg (\int _{B_{X,h}}\left| {\nabla u_h(Z) - R_{X_n,h}}\right| ^q \eta _X(Z) \ dZ\bigg ) \ dX\\&\le \sum _{n = 1}^{N_i} \int _{B_{X_n,h}}\bigg (\int _{B_{X_n,2h}}\left| {\nabla u_h(Z) - R_{X_n,h}}\right| ^q \eta _X(Z) \ dZ\bigg ) \ dX. \end{aligned}$$

Using Fubini’s theorem together with the fact that

$$\begin{aligned} \left| {\int _{B_{X_n,2h}} \eta _X(Z) \ dX}\right| \le \left\| {\eta _X}\right\| _{L^\infty (\hat{\Omega }_h)}\mathcal {L}^3(B_{X_n,2h}) \le C, \end{aligned}$$

by the Rigidity Estimate from Proposition (4.1), we conclude that:

$$\begin{aligned}&C \sum _{n = 1}^{N_i} \int _{B_{X_n,h}}\bigg (\int _{B_{X_n,2h}}\left| {\nabla u_h(Z) - R_{X_n,h}}\right| ^q \eta _X(Z) \ dZ\bigg ) \ dX\\&= C \sum _{n = 1}^{N_i} \int _{B_{X_n,2h}} \left| {\nabla u_h(Z) - R_{X_n,h}}\right| ^q \bigg (\int _{B_{X_n,h}} \eta _X(Z) \ dX\bigg ) \ dZ\\&\le C \sum _{n = 1}^{N_i} \int _{B_{X_n,2h}}\text {dist}^q(\nabla u(Z)_h, SO(3)) \ dZ\\&\le \tilde{C}\int _{\hat{\Omega }_h}\text {dist}^q(\nabla u_h(Z), SO(3)) \ dZ, \end{aligned}$$

as desired. The constant $\tilde{C}$ depends on the maximal covering number of $\hat{\Omega }_h$ by the family $(B_{X_n,2h})_{n = 1}^{N_i}$.

Using (4.12) together with (4.13), it follows that

$$\begin{aligned} \int _{\hat{\Omega }_h}\left| {\nabla u_h - R\pi _h}\right| ^q \ dX&\le C\bigg (\int _{\hat{\Omega }_h}\left| {\nabla u_h - \tilde{R}\pi _h}\right| ^q \ dX + \int _{\hat{\Omega }_h}\left| {R\pi _h -\tilde{R}\pi _h}\right| ^q \ dX\bigg )\\&= C\bigg (\int _{\hat{\Omega }_h}\left| {\nabla u_h - \tilde{R}\pi _h}\right| ^q \ dX + \int _{\hat{\Omega }_h}\text {dist}^q(\tilde{R}\pi _h, SO(3)) \ dX\bigg )\\&\le C\bigg (\int _{\hat{\Omega }_h}\left| {\nabla u_h - \tilde{R}\pi _h}\right| ^q \ dX + \int _{\hat{\Omega }_h}\text {dist}^q(\nabla u_h, SO(3)) \ dX\bigg )\\&\le C K(u_h, \hat{\Omega }_h), \end{aligned}$$

namely we deduce (i).

**Step 6** (Showing (iii)):

First an auxiliary matrix $\tilde{Q}$ is defined as the average of *R* over $\hat{\omega }_h$:By the Poincaré inequality and (ii) it follows that

4.14 $$\begin{aligned} \int _{\hat{\omega }_h}\left| {R(X) - \tilde{Q}}\right| ^q \ dX \le C \int _{\hat{\omega }_h}\left| {\hat{\nabla } R(X)}\right| ^q \ dX \le Ch^{-q-1}K(u_h,\hat{\Omega }_h). \end{aligned}$$

Arguing as in Step 5, we set $Q:= \Pi _{SO(3)}\tilde{Q}$, which is well defined due to the previous estimate. Since for any $X \in \hat{\omega }_h$ it holds that

$$\begin{aligned} \left| {\tilde{Q} - Q}\right| = \text {dist}(\tilde{Q}, SO(3)) \le \left| {\tilde{Q} - R(X)}\right| , \end{aligned}$$

by (4.14) we obtain

$$\begin{aligned} \int _{\hat{\omega }_h}\left| {R(X) - Q}\right| ^q \ dX&\le \int _{\hat{\omega }_h}\left| {R(X) - \tilde{Q}}\right| ^q + \left| {\tilde{Q} - Q}\right| ^q \ dX\\&\le 2 \int _{\hat{\omega }_h}\left| {R(X) - \tilde{Q}}\right| ^q \ dX\\&\le Ch^{-q-1}K(u_h,\hat{\Omega }_h), \end{aligned}$$

which concludes the proof. $\square$

By the change of variables from (3.10), we infer the following Corollary of Theorem 4.1:

#### Corollary 4.2

Let $y_h:= u_h \circ \Theta _h \circ z_h \in W^{1,p}(\Omega ;\mathbb {R}^3)$ and suppose that $h^{-p}K(u_h,\hat{\Omega }_h)$ is sufficiently small. Set

$$\begin{aligned} K_h(y_h,\Omega ) := \int _\Omega \text {dist}^2(\nabla _h y_h M_h, SO(3)) \vee \text {dist}^p(\nabla _h y_h M_h, SO(3)) \ dx. \end{aligned}$$

Then, there exist matrix fields $R_h \in W^{1,p}(\omega ;\mathbb {R}^{3\times 3})$ such that $R_h(x') \in SO(3)$ for all $x' \in \omega$, and matrices $Q_h \in SO(3)$ with the following properties:

4.15 $$\begin{aligned}&(i) \ \left\| {\nabla _h y_h M_h - R_h}\right\| _{L^q(\Omega ;\mathbb {R}^{3\times 3})}^q \le {CK_h(y_h,\Omega )}, \end{aligned}$$

4.16 $$\begin{aligned}&(ii) \ \left\| {\hat{\nabla } R_h}\right\| _{L^q(\omega ;\mathbb {R}^{3\times 3})}^q \le C{h^{-q}}K_h(y_h,\Omega ), \end{aligned}$$

4.17 $$\begin{aligned}&(iii) \ \left\| {R_h - Q_h}\right\| _{L^q(\omega ;\mathbb {R}^{3\times 3})}^q \le C{h^{-q}}K_h(y_h,\Omega ), \end{aligned}$$

where the constant *C* is independent of $y_h$ and *h*.

#### Proof

Set $R_h:= R \circ \pi _h \circ \Theta _h\circ z_h$ and let $Q_h$ be constructed as *Q* in the proof of Theorem 4.1. By the Change-of-Variables formula, Theorem 4.1 and (3.11), there holds

$$\begin{aligned}&\int _{\Theta _h\circ z_h(\Omega )}\left| {\nabla u_h - R\circ \pi _h}\right| ^q \ dX\\&= \int _\Omega \left| {((\nabla u_h) \circ \Theta _h\circ z_h) - R ( \pi _h(\Theta _h\circ z_h))}\right| ^q\left| {\det (\nabla \Theta _h\circ z_h)}\right| \ dx\\&= h\int _\Omega \left| {\nabla _h y_h M_h - R_h}\right| ^q\kappa _h \ dx\\&\le C \int _{\Theta _h\circ z_h(\Omega )}\text {dist}^2(\nabla u_h, SO(3)) \vee \text {dist}^p(\nabla u_h, SO(3)) \ dX\\&= Ch \int _\Omega \text {dist}^2(\nabla _h y_h M_h, SO(3)) \vee \text {dist}^p(\nabla _h y_h M_h, SO(3)) \ \kappa _h \ dx\\&\le ChK_h(y_h,\Omega ). \end{aligned}$$

Together with property (3.12), this shows (i).

For (*ii*) we first observe that the area-element of $\hat{\omega }_h$ is

$\left\| {\partial _1(\Theta _h \circ z_h (x')) \times \partial _2(\Theta _h \circ z_h (x'))}\right\| _2 = \sqrt{1 + h^2 \left\| {\nabla ' \theta (x')}\right\| ^2}$. With this we can conclude

$$\begin{aligned} \int _\omega \left| {(\nabla R_h) \circ \Theta _h \circ z_h}\right| ^q \ dx'&\le \int _\omega \left| {(\nabla R_h) \circ \Theta _h \circ z_h}\right| ^q\sqrt{1 + h^2 \left\| {\nabla ' \theta (x')}\right\| ^2} \ dx'\\&= \int _{\hat{\omega }_h}\left| {\hat{\nabla }R_h}\right| ^q \ dX \le Ch^{-q-1}K_h(u_h,\hat{\Omega }_h)\\&\le C h^{-q-1}h K_h(y_h,\Omega ). \end{aligned}$$

An analogous estimate shows (*iii*). $\square$

Utilizing (4.2) we obtain:

#### Corollary 4.3

Let $y_h \in W^{1,p}(\Omega ;\mathbb {R}^3)$. Suppose that $h^{-p}K(u_h,\hat{\Omega }_h)$ is sufficiently small and additionally that $E_h(y_h,m_h) \le C$ for every $h > 0$.

Then, there holds:

$$\begin{aligned}&(i) \ \left\| {\nabla _h y_h M_h - R_h}\right\| _{L^q(\Omega ;\mathbb {R}^{3\times 3})} \le Ch^{\frac{\beta }{q}},\\&(ii) \ \left\| {\nabla R_h}\right\| _{L^q(\omega ;\mathbb {R}^{3\times 3})} \le Ch^{\frac{\beta }{q} - 1},\\&(iii) \ \left\| {R_h - Q_h}\right\| _{L^q(\omega ;\mathbb {R}^{3\times 3})} \le Ch^{\frac{\beta }{q} - 1}, \end{aligned}$$

where the constant *C* is independent of $y_h$ and *h*.

We now use this approximation by rotations result to show compactness of deformations and magnetizations. The proofs of the following theorems are essentially adaptations of the analogous results from [1, Section 4] in the setting of plates.

### Compactness of Deformations

#### Proposition 4.4

(Compactness of deformations) Consider a sequence of admissible states $(y_h,m_h)_h \subseteq \mathcal {Q}$ satisfying $E_h(y_h, m_h) \le C$ for every $h > 0$. Then, there exists $u \in W^{1,2}(\omega ;\mathbb {R}^2)$, $v \in W^{2,2}(\omega )$, a sequence of rotations $(Q_h)_h \subseteq SO(3)$ and a sequence of translation vectors $(c_h)_h \subseteq \mathbb {R}^3$, such that by setting $\tilde{y}_h = T_h \circ y_h$, where $T_h: \mathbb {R}^3 \rightarrow \mathbb {R}^3$ is the rigid motion given by $T_h(\xi ):= Q_h^T\xi - c_h$ for every $\xi \in \mathbb {R}^3$, up to subsequences there holds:

$$\begin{aligned}&\tilde{y}_h \rightarrow \Theta _0 \circ z_0 := \lim _{h \rightarrow 0}\Theta _h\circ z_h \ \text {in} \ W^{1,p}(\Omega ;\mathbb {R}^3),\\&U_h^{\tilde{y}_h} \rightharpoonup u \ \text {weakly in} \ W^{1,2}(\omega ;\mathbb {R}^2) \ \text {for some} \ u \in W^{1,2}(\omega ;\mathbb {R}^2),\\&V_h^{\tilde{y}_h} \rightarrow v \ \text {in} \ W^{1,2}(\omega ) \ \text {for some} \ v \in W^{2,2}(\omega ). \end{aligned}$$

#### Proof

Choosing $R_h$ and $Q_h$ as in Corollary 4.3, we obtain the following estimates:

$$\begin{aligned}&\left\| {\nabla _h y_h M_h - R_h}\right\| _{L^q(\Omega ;\mathbb {R}^{3\times 3})} \le Ch^{\frac{\beta }{q}},\\&\left\| {R_h - Q_h}\right\| _{L^q(\omega ;\mathbb {R}^{3\times 3})} \le Ch^{\frac{\beta }{q} - 1}. \end{aligned}$$

Together this yields

4.18 $$\begin{aligned} \left\| {\nabla _h y_h M_h - Q_h}\right\| _{L^q(\Omega ;\mathbb {R}^{3\times 3})} \le Ch^{\frac{\beta }{q} - 1}. \end{aligned}$$

We setThen by consecutively applying the Poincaré-inequality, the chain rule, the fact that $\nabla \Theta _h\circ z_h$ is bounded by (2.4) and (4.18), we obtain

$$\begin{aligned} \left\| {\tilde{y}_h - \Theta _h\circ z_h}\right\| _{L^q(\Omega ;\mathbb {R}^{3\times 3})}&\le \left\| {\nabla \tilde{y}_h - \nabla (\Theta _h\circ z_h)}\right\| _{L^q(\Omega ;\mathbb {R}^{3\times 3})}\\&= \left\| {Q_h^T \nabla (u \circ \Theta _h\circ z_h) - \nabla (\Theta _h\circ z_h)}\right\| _{L^q(\Omega ;\mathbb {R}^{3\times 3})}\\&= \left\| {Q_h^T ((\nabla u) \circ \Theta _h\circ z_h)\nabla (\Theta _h \circ z_h) - \nabla (\Theta _h\circ z_h)}\right\| _{L^q(\Omega ;\mathbb {R}^{3\times 3})}\\&\le C \left\| {Q_h^T\nabla _h y_h M_h - Id}\right\| _{L^q(\Omega ;\mathbb {R}^{3\times 3})}\\&\le Ch^{\frac{\beta }{q}}, \end{aligned}$$

where we used the fact that,

$$\begin{aligned} \left\| {\nabla (\Theta _h \circ z_h)}\right\| _{L^\infty (\Omega ;\mathbb {R}^{3\times 3})} \le C. \end{aligned}$$

Since $$\beta > 2p$$, $$q \in \{2,p\}$$ and

$$\begin{aligned} \frac{\beta }{q} \ge \frac{\beta }{p} > 2, \end{aligned}$$

it follows that $\tilde{y}_h \rightarrow \Theta _0 \circ z_0 \ \text {in} \ W^{1,p}(\Omega ;\mathbb {R}^3)$, as $h \rightarrow 0^+$.

We obtain $U_h^{\tilde{y}_h} \rightharpoonup u \ \text {weakly in} \ W^{1,2}(\omega ;\mathbb {R}^2)$ and $V_h^{\tilde{y}_h} \rightarrow v \ \text {in} \ W^{1,2}(\omega )$ from equations (32) and (33) in [18, Lemma 2]. $\square$

### Compactness of Magnetizations

#### Proposition 4.5

(Compactness of Magnetizations). For a sequence of admissible maps $((y_h, m_h))_h \subseteq \mathcal {Q}$ with $E_h^{el}(y_h,m_h) + E_h^{exc}(y_h,m_h) \le C$, for every $h > 0$ we set $\tilde{y}_h:= T_h \circ y_h$ and $\tilde{m}_h:= Q_h^Tm_h\circ T_h^{-1}$, with $Q_h \in SO(3)$ and $T_h: \mathbb {R}^3 \rightarrow \mathbb {R}^3$ defined as in Theorem 4.4. Then, there exists a map $\lambda \in W^{1,2}(\omega ;\mathbb {S}^2)$, such that, up to subsequences, there holds:

4.19 $$\begin{aligned}&\tilde{m}_h \circ \tilde{y}_h \rightarrow \lambda \ \ \text {in} \ \ L^r(\Omega ;\mathbb {R}^3) \ \ \text {for every} \ 1\le r < \infty , \end{aligned}$$

4.20 $$\begin{aligned}&\tilde{\mu }_h := (\chi _{\Omega ^{\tilde{y}_h}}m_h)\circ \Theta _h\circ z_h \rightarrow \chi _{\Omega }\lambda \ \ \text {in} \ \ L^r(\mathbb {R}^3;\mathbb {R}^3) \ \ \text {for every} \ 1\le r < \infty . \end{aligned}$$

#### Proof

The proof is an adaptation of [1, Proposition 4.2], for shallow shells. It follows the same four steps approach. Steps 1 and 2 require arguments adapted for shallow shells and are presented, whereas Steps 3 and 4 require no adaptation and are therefore obmitted.

**Step 1** (Existence of an inner cylinder):

From Theorem 4.4 we know that

$$\begin{aligned} \left\| {\tilde{y}_h - \Theta _h\circ z_h}\right\| _{L^p(\Omega ;\mathbb {R}^{3\times 3})} \le Ch^{\frac{\beta }{p}} := \gamma _h, \end{aligned}$$

and by Sobolev embeddings,

$$\begin{aligned} \left\| {\tilde{y}_h - \Theta _h\circ z_h}\right\| _{C^0(\overline{\Omega };\mathbb {R}^3)} \le \gamma _h. \end{aligned}$$

Now, define for $a, b > 0$

$$\begin{aligned}&\omega ^a := \{ x' \in \omega \ : \ \text {dist}(x',\partial \omega ) > a \},\\&\Omega ^a_b := \omega ^a \times \big (-\frac{b}{2},\frac{b}{2}\big ),\\&\omega ^{-a} := \{ x' \in \mathbb {R}^2 \ : \ \text {dist}(x',\partial \omega ) < a \},\\&\Omega ^{-a}_b := \omega ^{-a} \times \big (-\frac{b}{2},\frac{b}{2}\big ). \end{aligned}$$

Then, we set $\hat{\Omega }_{bh}^a = \Theta _h(z_h(\Omega ^a_{b}))$ and $\hat{\Omega }_{bh}^{-a} = \Theta _h(z_h(\Omega _b^{-a}))$.

In the next argument we write $\text {dist}_{\hat{\omega }_h}$ for the geodesic distance on $\hat{\omega }_h$ and use that since $\Theta _h \circ z_h$ is a $C^1$-Diffeomorphism, it is a bi-Lipschitz map.

We fix a $\varepsilon > 0$ and $\tau \in (0,1)$. Then for every $x \in \Omega _{\tau }^\varepsilon$, using the fact that $\Theta _h$ is bi-Lipschitz on $\overline{\Omega }_h$ and the definition of $\Omega _{\tau }^\varepsilon$, there holds

$$\begin{aligned} \text {dist}(\Theta _h(z_h(x)), \partial \hat{\Omega }_h)&\ge C\text {dist}(x,\partial \Omega _h)\\&\ge C (\text {dist}(x',\partial \omega ) \wedge h(\frac{1}{2} - \left| {x_3}\right| ))\\&> C(\varepsilon \wedge h\frac{1 - \tau }{2}). \end{aligned}$$

Recalling $\gamma _h = Ch^{\frac{\beta }{p}}$ and $\frac{\beta }{p} > 2$, we observe that for sufficiently small *h* there holds

$$C(\varepsilon \wedge h\frac{1 - \tau }{2}) > \gamma _h$$, which implies

$$\begin{aligned} \left\| {\tilde{y}_h - \Theta _h}\right\| _{C^0(\overline{\Omega };\mathbb {R}^3)} < \text {dist}(\Theta _h(z_h(x)), \partial \hat{\Omega }_h). \end{aligned}$$

From this, it follows by [1, Proposition 4.2 Step 1] that

4.21 $$\begin{aligned} \forall \ \varepsilon> 0, \forall \ \tau \in (0,1), \exists \ \overline{h}(\varepsilon ,\tau ) > 0: \forall \ h \in (0,\overline{h}(\varepsilon ,\tau )], \ \hat{\Omega }^\varepsilon _{\tau h} \subseteq \Omega ^{\tilde{y}_h}. \end{aligned}$$

**Step 2** (Convergence of magnetizations):

By the chain rule, there holds

$$\begin{aligned} \nabla \tilde{m}_h = Q_h^T\nabla m_h \circ T_h^{-1}Q_h, \end{aligned}$$

and applying the Change-of-Variable formula, we obtain

$$\begin{aligned} \begin{aligned} E_h^{exc}(\tilde{y}_h, \tilde{m}_h)&= \frac{\alpha }{h} \int _{\Omega ^{\tilde{y}_h}}\left| {\nabla \tilde{m}_h}\right| ^2 \ d\xi = \frac{\alpha }{h}\int _{T_h(\Omega ^{y_h})}\left| {\nabla m_h \circ T_h^{-1}}\right| ^2 \ d\xi \\&= \frac{\alpha }{h}\int _{\Omega ^{y_h}}\left| {\nabla m_h}\right| ^2 \ d\xi = E_h^{exc}(y_h, m_h). \end{aligned} \end{aligned}$$

Now we fix $\varepsilon > 0$ and $\tau \in (0,1)$ and apply (4.21), so that for $h < \overline{h}(\varepsilon ,\tau )$ we infer $\hat{\Omega }^\varepsilon _{\tau h} \subseteq \Omega ^{\tilde{y}_h}$. Using this, we obtain

$$\begin{aligned} C \ge E_h^{exc}(\tilde{y}_h, \tilde{m}_h)&= \frac{\alpha }{h}\int _{\Omega ^{\tilde{y}_h}}\left| {\nabla \tilde{m}_h}\right| ^2 \ d\xi \\&\ge \frac{\alpha }{h}\int _{\hat{\Omega }^\varepsilon _{\tau h}}\left| {\nabla \tilde{m}_h}\right| ^2 \ d\xi . \end{aligned}$$

Also, $\tilde{m}_{h_{|\hat{\Omega }^\varepsilon _{\tau h}}}$ or analogously

$$\begin{aligned} \hat{m}_h := \tilde{m}_h \circ \Theta _h \circ z_h(\Omega ^\varepsilon _\tau ), \end{aligned}$$

is well-defined and a map in $W^{1,2}(\Omega ^\varepsilon _\tau ; \mathbb {S}^2)$. By a straightforward calculation for $x \in \Omega ^\varepsilon _\tau$ and $\xi = (\Theta _h\circ z_h) (x)$ with the notation from (3.9), we obtain

$$\begin{aligned} (\nabla \tilde{m}_h)(\xi )&= (\nabla \tilde{m}_h) \circ \Theta _h \circ z_h (x)\\&= ((\nabla \Theta _h)^{-T}\circ z_h)(x)(\nabla (\tilde{m}_h\circ \Theta _h)\circ z_h)(x)\\&= M_h^T(x)(\nabla _h(\tilde{m}_h \circ \Theta _h \circ z_h))(x)\\&= M_h^T(x)(\nabla _h\hat{m}_h)(x)\\&= (M_h^T \circ (\Theta _h\circ z_h)^{-1})(\xi )(\nabla _h\hat{m}_h\circ (\Theta _h\circ z_h)^{-1})(\xi ). \end{aligned}$$

Using this identity together with $\left| {(M_h^T \circ (\Theta _h\circ z_h)^{-1})(\xi )}\right| = \left| {M_h^T(x)}\right|$, property (3.14) and the Change-of-Variable formula, we infer

$$\begin{aligned} \frac{\alpha }{h}\int _{\hat{\Omega }^\varepsilon _{\tau h}}\left| {\nabla \tilde{m}_h}\right| ^2 \ d\xi&= \frac{\alpha }{h}\int _{\hat{\Omega }^\varepsilon _{\tau h}}\left| {((\nabla \Theta _h)^{-T}\circ z_h \circ (\Theta _h\circ z_h)^{-1})\nabla _h\hat{m}_h\circ (\Theta _h\circ z_h)^{-1}}\right| ^2 \ d\xi \\&\ge C\frac{\alpha }{h}\int _{\hat{\Omega }^\varepsilon _{\tau h}}\left| {(\nabla _h\hat{m}_h\circ (\Theta _h\circ z_h)^{-1})}\right| ^2 \ d\xi \\&= C\frac{\alpha }{h}\int _{\Omega ^\varepsilon _\tau }\left| {\nabla _h \hat{m}_h}\right| ^2\left| {\det \nabla (\Theta _h \circ z_h)}\right| \ dx\\&\ge C\alpha \int _{\Omega ^\varepsilon _\tau }\left| {\nabla _h \hat{m}_h}\right| ^2 \ dx. \end{aligned}$$

From this estimate it firstly follows with

$$\begin{aligned} \left\| {\nabla \hat{m}_h}\right\| _{L^2(\Omega ^\varepsilon _\tau ;\mathbb {R}^3)} \le \left\| {\nabla _h \hat{m}_h}\right\| _{L^2(\Omega ^\varepsilon _\tau ;\mathbb {R}^3)} \le C, \end{aligned}$$

that

$$\begin{aligned} \hat{m}_h \rightharpoonup \lambda \ \ \text {in} \ \ W^{1,2}(\Omega ^\varepsilon _\tau ;\mathbb {R}^3), \end{aligned}$$

for some $\lambda \in W^{1,2}(\Omega ^\varepsilon _\tau ;\mathbb {R}^3)$ and secondly from

$$\begin{aligned} \left\| {h^{-1}\partial _3 \hat{m}_h}\right\| _{L^2(\Omega ^\varepsilon _\tau ;\mathbb {R}^3)} \le C, \end{aligned}$$

that

$$\begin{aligned}&\partial _3 \hat{m}_h \rightharpoonup 0 \ \ \text {in} \ \ L^2(\Omega ^\varepsilon _\tau ;\mathbb {R}^3),\\&h^{-1}\partial _3 \hat{m}_h \rightharpoonup k \ \ \text {in} \ \ L^2(\Omega ^\varepsilon _\tau ;\mathbb {R}^3), \end{aligned}$$

for some $$ k \in L^2(\Omega ^\varepsilon _\tau ;\mathbb {R}^3)$$.

Since the bound for $\left\| {\nabla \hat{m}_h}\right\| _{L^2(\Omega ^\varepsilon _\tau ;\mathbb {R}^3)}$ is independent of $\varepsilon$ and $\tau$, so is the convergence. Together with the fact that $\lambda$ does not depend on the third component $x_3$, it follows that $\lambda \in W^{1,2}_{loc}(\omega ;\mathbb {S}^2)$ and $k \in L^2_{loc}(\Omega ;\mathbb {R}^3)$. Additionally, by lower semi-continuity of the norm it follows that

$$\begin{aligned} C&\ge \liminf _{h \rightarrow 0^+}\int _{\Omega ^\varepsilon _\tau }\left| {\nabla _h \hat{m}_h}\right| ^2 \ dx \ge \int _{\Omega ^\varepsilon _\tau }\left| {(\nabla '\lambda , k)}\right| ^2 \ dx\\&\ge \int _{\Omega ^\varepsilon _\tau }\left| {\nabla '\lambda }\right| ^2 \ dx + \int _{\Omega ^\varepsilon _\tau }\left| {k}\right| ^2 \ dx \ge \tau \int _{\omega ^\varepsilon }\left| {\nabla '\lambda }\right| ^2 \ dx. \end{aligned}$$

Therefore by letting $\varepsilon \rightarrow 0^+$ and $\tau \rightarrow 1^-$ it follows that $\nabla '\lambda \in L^2(\omega ;\mathbb {R}^2)$, so that $\lambda \in W^{1,2}(\omega ;\mathbb {S}^2)$.

From this, by [1, Proposition 4.2 Step 3 and Step 4] the convergences in (4.19) and (4.20) follow. $\square$

## Lower Bound and Recovery Sequence

This section is dedicated to the second part of the proof of Theorem 3.4 and to the proof of Theorem 3.5. We adapt arguments of [1, Section 4] when necessary.

### Proposition 5.1

(Lower bound for the exchange energy). Let $((y_h,m_h))_h \subseteq \mathcal {Q}$, be such that $E^{el}_h(y_h,m_h) + E^{exc}_h(y_h,m_h) \le C$, for all $h >0$. Let $\lambda \in W^{1,2}(\omega ; \mathbb {S}^2)$ be as in Theorem 4.5. Then,

$$\begin{aligned} E^{exc}(\lambda ) \le \liminf _{h \rightarrow 0^+}E^{exc}_h(y_h,m_h). \end{aligned}$$

### Proof

The proof is a simple adaptation of [1, Proposition 4.3] using Proposition 4.5.

$$\square $$

### Proposition 5.2

(Lower bound for the elastic energy) Let $((y_h,m_h))_h \subseteq \mathcal {Q}$ be such that $E^{el}_h(y_h,m_h) + E^{exc}_h \le C$ for all $h > 0$. Then, for $u \in W^{1,2}(\omega ;\mathbb {R}^2)$, $v \in W^{2,2}(\omega )$ and $\lambda \in W^{1,2}(\omega ;\mathbb {S}^2)$ as in Theorem 4.4 and Theorem 4.5, there holds

$$\begin{aligned} E^{el}(u,v,\lambda ) \le \liminf _{h\rightarrow 0^+}E^{el}_h(y_h,m_h). \end{aligned}$$

### Proof

As in Theorem 4.4, we set $\tilde{y}_h:= T_h \circ y_h$ and $\tilde{m}_h:= Q_h^Tm_h\circ T_h^{-1}$ and we define

$$ F_h:= \nabla _h y_h M_h, \quad \tilde{F}_h:= Q_h^TF_h, \quad \tilde{R}_h:= Q_h^TR_h, $$

where $R_h$ is given by Corollary 4.3. Setting

$$ G_h:= \frac{1}{h^{\frac{\beta }{2}}}(\tilde{R}_h^T\tilde{F}_h - Id), $$

by [18, (59) and (60)], we obtain

$$\begin{aligned} G_h \rightharpoonup G \ \ \text {in} \ L^2(\Omega ;\mathbb {R}^{3\times 3}), \end{aligned}$$

with

5.1 $$\begin{aligned} G''(x)&= K(x') + L(x')x_3, \end{aligned}$$

for almost every $x \in \Omega$, where $K:= \text {sym}(\nabla 'u + \nabla 'v \otimes \nabla '\theta ) \in \mathbb {R}^{2\times 2}$ and $L:= -(\nabla ')^2v \in \mathbb {R}^{2\times 2}$. Here *u* and *v* are the averaged limiting displacements identified in (3.18) and (3.19).

The proof concludes analogously to the one of [1, Proposition 4.4]. $\square$

### Proposition 5.3

(Convergence of the magnetostatic energy). Let $((y_h,m_h))_h \subseteq \mathcal {Q}$ be such that $E_h(y_h,m_h) \le C$ for every $h > 0$. Let $\lambda \in W^{1,2}(\omega ;\mathbb {S}^2)$ be the map identified in Theorem 4.5. Then, up to subsequences, there holds:

$$\begin{aligned} \lim _{h\rightarrow 0^+}E_h^{mag}(y_h,m_h) = E^{mag}(\lambda ). \end{aligned}$$

### Proof

The proof is a simple adaptation of [1, Proposition 4.5] using Lemma 3.2 to obtain bounds for the shallow shell map $\Theta _h \circ z_h$ whenever it occurs. $\square$

### Recovery Sequence

We follow the strategy in [1, Section 5] of first constructing a smooth recovery sequence and then arguing by density to obtain the desired recovery sequence.

#### Proposition 5.4

(Smooth recovery sequence). Let $u \in C^\infty (\overline{\omega };\mathbb {R}^2)$, $v \in C^\infty (\overline{\omega })$ and $\lambda \in C^\infty (\overline{\omega };\mathbb {S}^2)$. Let $a, b \in C^\infty (\overline{\omega };\mathbb {R}^3)$ satisfy

$$\begin{aligned} \begin{aligned}&\text {tr}\bigg (\left( \begin{array}{c|c} \text {sym}(\nabla 'u + \nabla ' v \otimes \nabla ' \theta ) &  0'\\ \hline (0')^T &  0 \end{array}\right) + a \otimes e_3 + e_3 \otimes a \bigg )\\ = \  &\text {div}'(u) + \nabla 'v \cdot \nabla '\theta + 2 (a)^3 = 0 \ \ \text {in} \ \omega , \end{aligned} \end{aligned}$$

$$\begin{aligned} \begin{aligned}&\text {tr}\bigg (-\left( \begin{array}{c|c} (\nabla ')^2v &  0'\\ \hline (0')^T &  0 \end{array}\right) + b \otimes e_3 + e_3 \otimes b \bigg )\\ = \  &-\Delta 'v + 2 (b)^3 = 0 \ \ \text {in} \ \omega . \end{aligned} \end{aligned}$$

Then, there exists a sequence of admissible states $((y_h,m_h))_h \subseteq \mathcal {Q}$ such that, as $h \rightarrow 0^+$:

$$\begin{aligned}&y_h \rightarrow \Theta _0 \circ z_0 \ \ \text {in} \ W^{1,p}(\Omega ;\mathbb {R}^3),\\&u_h := U_h^{y_h} \rightarrow u \ \ \text {in} \ W^{1,2}(\omega ;\mathbb {R}^2),\\&v_h := V_h^{y_h} \rightarrow v \ \ \text {in} \ W^{1,2}(\omega ), \\&m_h \circ y_h \rightarrow \lambda \ \ \text {in} \ L^{r}(\mathbb {R}^3;\mathbb {R}^3) \ \text {for every} \ 1 \le r< \infty ,\\&\mu _h := (\chi _{\Omega ^{y_h}}m)\circ \Theta _h \circ z_h \rightarrow \chi _\Omega \lambda \ \ \text {in} \ L^{r}(\Omega ;\mathbb {R}^3) \ \text {for every} \ 1 \le r < \infty . \end{aligned}$$

Moreover, there holds:

$$\begin{aligned} \begin{aligned}&\lim _{h \rightarrow 0^+}E_h^{el}(y_h,m_h)\\&= \frac{1}{2}\int _\omega Q_2^{inc}\bigg (\left( \begin{array}{c|c} \text {sym}(\nabla ' u + \nabla 'v \otimes \nabla '\theta ) &  0'\\ \hline (0')^T &  0 \end{array}\right) + a \otimes e_3 + e_3 \otimes a, \lambda \bigg ) \ dx'\\&+ \frac{1}{24}\int _\omega Q_2^{inc}\bigg (-\left( \begin{array}{c|c} (\nabla ')^2v &  0'\\ \hline (0')^T &  0 \end{array}\right) + b \otimes e_3 + e_3 \otimes b, \lambda \bigg ) \ dx'; \end{aligned} \end{aligned}$$

$$\begin{aligned}&\lim _{h \rightarrow 0^+}E_h^{exc}(y_h,m_h) = E^{exc}(\lambda );\\&\lim _{h \rightarrow 0^+}E_h^{mag}(y_h,m_h) = E^{mag}(\lambda ); \end{aligned}$$

#### Proof

We adapt the proof of [1, Proposition 5.1] for incompressible magnetoelastic shallow shells.

**Step 1** (Gradient of the recovery sequence):

We make the following ansatz for the recovery sequence:

$$\begin{aligned} \begin{aligned} \overline{y}_h := \  &\Theta _h\circ z_h + h^{\frac{\beta }{2}}\begin{pmatrix}u\\ 0\end{pmatrix} + h^{\frac{\beta }{2}-1}\begin{pmatrix}0'\\ v\end{pmatrix} - h^{\frac{\beta }{2}}x_3\begin{pmatrix}\nabla 'v\\ 0\end{pmatrix}\\&+ 2h^{\frac{\beta }{2}+1}x_3\tilde{a} + 2h^{\frac{\beta }{2}+1}x_3^2b. \end{aligned} \end{aligned}$$

Using the fact that $\nabla \overline{y}_h = \nabla _h \overline{y}_h \nabla z_h$, we obtain

$$\begin{aligned} \nabla _h \overline{y}_h M_h = \nabla \overline{y}_h (\nabla z_h)^{-1} M_h. \end{aligned}$$

Further, there holds

$$\begin{aligned} (\nabla z_h)^{-1} M_h&= (\nabla z_h)^{-1}((\nabla \Theta _h)\circ z_h)^{-1} = ((\nabla \Theta _h) \circ z_h \nabla z_h)^{-1} = (\nabla (\Theta _h \circ z_h))^{-1}. \end{aligned}$$

Together, this yields

5.2 $$\begin{aligned} \nabla _h \overline{y}_h M_h = \nabla \overline{y}_h (\nabla (\Theta _h \circ z_h))^{-1}. \end{aligned}$$

Next, we calculate $\nabla _h \overline{y}_h M_h$ up to order $h^{\frac{\beta }{2}}$. We use (5.2) for the first term to obtain

$$\begin{aligned} \nabla _h(\Theta _h \circ z_h) M_h = \nabla (\Theta _h \circ z_h) (\nabla (\Theta _h \circ z_h))^{-1} = Id. \end{aligned}$$

Recall that by (2.5), there holds

$$\begin{aligned} M_h = Id + h A \pi + h^2 G_h \circ z_h, \end{aligned}$$

with $$A(x') = \begin{pmatrix} 0 &  0 &  \partial _1\theta (x')\\ 0 &  0 &  \partial _2\theta (x')\\ -\partial _1\theta (x') &  -\partial _2\theta (x') &  0 \end{pmatrix}$$.

Using this we obtain

5.3 $$\begin{aligned} \begin{aligned} \nabla _h \overline{y}_h M_h = \  &Id + h^{\frac{\beta }{2}}\left( \begin{array}{c|c} \nabla ' u + \nabla 'v \otimes \nabla '\theta &  0'\\ \hline (0')^T &  0 \end{array}\right) \\ +&h^{\frac{\beta }{2}-1}\left( \begin{array}{c|c} 0'' &  -\nabla 'v\\ \hline \nabla 'v^T &  0 \end{array}\right) - h^{\frac{\beta }{2}}x_3 \left( \begin{array}{c|c} (\nabla ')^2 v &  0'\\ \hline (0')^T &  0 \end{array}\right) \\ +&2h^{\frac{\beta }{2}}a \otimes e_3 + 2 h^{\frac{\beta }{2}}x_3 b \otimes e_3 + \mathcal {O}(h^{\frac{\beta }{2}+1}), \end{aligned} \end{aligned}$$

where $$a:= \tilde{a} + ((0')^T, \nabla ' v \cdot \nabla ' \theta )^T$$.

**Step 2** (Incompressibility constraint):

By the incompressibility constraint (3.2), $F_h:= \nabla _h y_h M_h$ needs to satisfy $\det (F_h) = 1$. To achieve this, we proceed as follows:

Call $\overline{F}_h:= \nabla _h \overline{y}_h M_h$ and write $\overline{F}_h = Id + G_h$.

With the fact that

$$\begin{aligned} (Id + G_h)^T(Id + G_h) = Id + 2 \text {sym}G_h + G_h^TG_h, \end{aligned}$$

together with $G_h^TG_h = \mathcal {O}(h^{\frac{\beta }{2}+1})$, as $\frac{\beta }{2} > 3$, we conclude with (5.3) by a calculation that

5.4 $$\begin{aligned} \overline{F}_h^T\overline{F}_h = Id + 2 h^{\frac{\beta }{2}}(A + x_3 B) + O(h^{\frac{\beta }{2} +1}), \end{aligned}$$

with

$$\begin{aligned} A&= \left( \begin{array}{c|c} \text {sym}(\nabla ' u + \nabla 'v \otimes \nabla '\theta ) &  0'\\ \hline (0')^T &  0 \end{array}\right) + a \otimes e_3 + e_3 \otimes a,\\ B&= -\left( \begin{array}{c|c} (\nabla ')^2v &  0'\\ \hline (0')^T &  0 \end{array}\right) + b \otimes e_3 + e_3 \otimes b. \end{aligned}$$

Analogous to [1, Proposition 5.1], we use the fact that for every $M \in \mathbb {R}^{3\times 3}$ there holds

$$\begin{aligned} \det (Id + M) = 1 + \text {tr}(M) + \text {tr}(\text {cof}(M)) + \det (M), \end{aligned}$$

to conclude by (5.4) together with the assumption $\text {tr}(A) = \text {tr}(B) = 0$ that

$$\begin{aligned} \det (\overline{F}_h^T\overline{F}_h) = 1 + 2h^{\beta }(P + x_3Q + x_3^2R) + \mathcal {O}(h^{\frac{\beta }{2}+1}), \end{aligned}$$

where *P*, *Q* and *R* are polynomials in $x'$ which depend on $u, v, \theta , a$ and *b*.

Using $\det (\overline{F}_h^T\overline{F}_h) = \det (\overline{F}_h)^2$ together with the Taylor expansion

$$(1 + 2x)^{\frac{1}{2}} = 1 + x + \mathcal {O}(x^2)$$, we infer

5.5 $$\begin{aligned} \det (\overline{F}_h) = 1 + h^\beta (P + x_3Q + x_3^2R) + \mathcal {O}(h^{\frac{\beta }{2}+1}). \end{aligned}$$

We define $y_h(x',x_3):= \overline{y}_h(x',\eta _h(x', x_3))$ for $x \in \Omega$, for a function $\eta _h$ which we will specify later on and set $F_h:= \nabla _h y_h M_h$. By writing this in terms of the map $\phi : (x',x_3) \mapsto (x', \eta _h(x',x_3))$ there holds

$$\begin{aligned} F_h&= \nabla _h y_h M_h\\&= ((\nabla _h \overline{y}_h) \circ \phi )M_h M_h^{-1} \nabla \phi M_h. \end{aligned}$$

With $$\nabla \phi = \begin{pmatrix} 1 &  0 &  0\\ 0 &  1 &  0\\ \partial _1\eta _h &  \partial _2\eta _h &  \partial _3\eta _h \end{pmatrix}$$, we conclude that

$$\begin{aligned} \det (M_h^{-1} \nabla \phi M_h) = \det (\nabla \phi ) = \partial _3 n_h. \end{aligned}$$

To summarize, we have shown that for all $x \in \Omega$ there holds

5.6 $$\begin{aligned} \begin{aligned}&F_h(x',x_3) = \overline{F}_h(x',\eta _h(x',x_3)) M_h^{-1}(x',x_3) \nabla \phi (x',x_3) M_h (x',x_3)\\&\det (F_h(x',x_3)) = \det (\overline{F}_h(x', \eta _h(x',x_3)))\partial _3 \eta _h(x',x_3). \end{aligned} \end{aligned}$$

Following [1, (5.17)], by the fact that $\beta > 2$ and (5.5), there holds $\det (\overline{F}_h) = 1 + \mathcal {O}(h^{\frac{\beta }{2} + 1})$ and therefore $\frac{1}{2} \le \det (\overline{F}_h) \le 2$ for *h* small enough. With this, we can define

$$\begin{aligned} \Phi _h(x',x_3) := (\det (\overline{F}_h(x',x_3)))^{-1} = (1 + h^\beta (P + x_3Q + x_3^2R) + \mathcal {O}(h^{\frac{\beta }{2}+1}))^{-1}, \end{aligned}$$

for every $(x',x_3) \in \omega \times (-1,1)$. By (5.6), if $\eta _h$ is a solution of the Cauchy problem

5.7 $$\begin{aligned} \left\{ \begin{aligned} \partial _3 \eta _h(x',x_3)&= \Phi _h(x',\eta _h(x',x_3)), \ \text {in} \ \big (-\frac{1}{2}, \frac{1}{2}\big )\\ \eta _h(x',0)&= 0, \end{aligned} \right. \end{aligned}$$

then $\det (\nabla _h y_h M_h) = \det (F_h) = 1$ in $\Omega$. By regarding $x' \in \omega$ as a parameter and $x_3 \in (-\frac{1}{2}, \frac{1}{2})$ as a variable, (5.7) is an ordinary differential equation.

From [1, Proposition 5.1, Steps 2 and 3], we obtain the following two results:

There exists a unique solution $\eta _h$ of (5.7) satisfying the estimates

5.8 $$\begin{aligned} \left\| {\partial _3 \eta _h - 1}\right\| _{C^0(\overline{\Omega })} \le Ch^{\frac{\beta }{2}+1}, \ \left\| {\eta _h - x_3}\right\| _{C^0(\overline{\Omega })} \le Ch^{\frac{\beta }{2}+1}, \ \left\| {\nabla '\eta _h}\right\| _{C^0(\overline{\Omega })} \le Ch^{\frac{\beta }{2}+1}, \end{aligned}$$

and there holds

$$\begin{aligned} F_h^T(x)F_h(x) = \overline{F}_h^T(x)\overline{F}_h(x) + \mathcal {O}(h^{\frac{\beta }{2} + 1}). \end{aligned}$$

**Step 3** (Injectivity of the recovery sequence):

By construction, $y_h \in C^1(\overline{\Omega };\mathbb {R}^3)$ and satisfies $\det (\nabla y_h) = \det \nabla (\Theta _h \circ z_h) = h\kappa _h$ by the incompressibilty constraint. Recall that by the ansatz for $\overline{y}_h$ we have

$$\begin{aligned} \begin{aligned} y_h = \  &\Theta _h \circ z_h \circ \phi + h^{\frac{\beta }{2}}\begin{pmatrix}u\\ 0\end{pmatrix} + h^{\frac{\beta }{2}-1}\begin{pmatrix}0'\\ v\end{pmatrix} - h^{\frac{\beta }{2}}\eta _h\begin{pmatrix}\nabla 'v\\ 0\end{pmatrix}\\&+ 2h^{\frac{\beta }{2}+1}\eta _ha + 2h^{\frac{\beta }{2}+1}\eta _h^2b. \end{aligned} \end{aligned}$$

Setting $\varphi _h:= y_h - \Theta _h \circ z_h$, we compute

$$\begin{aligned} \begin{aligned} \nabla _h\varphi _h = \  &S + h^{\frac{\beta }{2}}\left( \begin{array}{c|c} \nabla ' u + \nabla 'v \otimes \nabla '\theta &  0'\\ \hline (0')^T &  0 \end{array}\right) \\&+ h^{\frac{\beta }{2}-1}e_3 \otimes \begin{pmatrix}\nabla 'v\\ 0\end{pmatrix} - h^{\frac{\beta }{2}}\begin{pmatrix}\nabla 'v\\ 0\end{pmatrix} \otimes \nabla _h\eta _h - h^{\frac{\beta }{2}}\eta _h \left( \begin{array}{c|c} (\nabla ')^2 v &  0'\\ \hline (0')^T &  0 \end{array}\right) \\&+ 2h^{\frac{\beta }{2}}a \otimes \nabla _h\eta _h + 2h^{\frac{\beta }{2}+1}\eta _h\left( \begin{array}{c|c} \nabla 'a&0\end{array}\right) \\&+ 2 h^{\frac{\beta }{2}}\eta _h b \otimes \nabla _h\eta _h + 2h^{\frac{\beta }{2}+1}\eta _h^2\left( \begin{array}{c|c} \nabla 'b&0\end{array}\right) , \end{aligned} \end{aligned}$$

where, recalling that for $x \in \Omega$, $\Theta _h(z_h(x)) = (x',h \theta (x')) + hx_3n_h(x')$ we wrote

$$\begin{aligned} S :=&\ \nabla _h (\Theta _h \circ z_h \circ \phi - \Theta _h \circ z_h)\\ =&\ h\left( \begin{array}{c|c|c} \partial _1 n_h (\eta _h - x_3) + n_h\partial _1 \eta _h&\partial _2 n_h (\eta _h - x_3) + n_h\partial _2 \eta _h&\frac{1}{h} n_h(\partial _3 \eta - 1)\end{array}\right) . \end{aligned}$$

Using that by Proposition 2.5, $\partial _in_h$ is bounded by some constant and the estimates (5.8), we obtain

5.9 $$\begin{aligned} \left\| {\nabla _h \varphi _h}\right\| _{C^0(\overline{\Omega };\mathbb {R}^{3 \times 3})} \le Ch^{\frac{\beta }{2} - 1}. \end{aligned}$$

Setting $\psi _h:= \varphi _h \circ (\Theta _h \circ z_h)^{-1}_{|\hat{\Omega }_h}$ together with the fact that $\nabla \Theta ^{-1}$ is bounded (see (2.5)), we obtain

$$\begin{aligned} \begin{aligned} \left\| {\nabla \psi _h}\right\| _{C^0(\overline{\hat{\Omega }}_h;\mathbb {R}^{3 \times 3})}&= \left\| {\nabla \varphi _h \circ (\Theta _h \circ z_h)^{-1} \nabla (\Theta _h \circ z_h)^{-1}}\right\| _{C^0(\overline{\hat{\Omega }}_h;\mathbb {R}^{3 \times 3})}\\&= \left\| {\nabla \varphi _h \circ (\Theta _h \circ z_h)^{-1} (\nabla z_h^{-1}\nabla z_h) \nabla (\Theta _h \circ z_h)^{-1}}\right\| _{C^0(\overline{\hat{\Omega }}_h;\mathbb {R}^{3 \times 3})}\\&= \left\| {\nabla _h \varphi _h \circ (\Theta _h \circ z_h)^{-1} \nabla z_h \nabla (\Theta _h \circ z_h)^{-1}}\right\| _{C^0(\overline{\hat{\Omega }}_h;\mathbb {R}^{3 \times 3})}\\&\le Ch^{\frac{\beta }{2}-1}. \end{aligned} \end{aligned}$$

With this, analogously to [1, Proposition 5.1 Step 4] using the same arguments from [7, Theorem 5.5-1], injectivity of $y_h$ follows.

Then [1, Proposition 5.1 Step 5 and Step 6] finish proof by providing the necessary convergences and require no adaptation. $\square$

With the previous result, the proof of Theorem 3.5 follows immediately.

#### Proof of Theorem 3.5

Using Proposition 5.4, we conclude by the same density argument as conducted in the proof of [1, Theorem 3.2]. $\square$

## Data Availability

No datasets were analyzed during the current study.
